# Novel Mechanism of and Therapeutic Approach for Anthracycline-Induced Cardiotoxicity

**DOI:** 10.1158/2767-9764.CRC-25-0511

**Published:** 2026-06-01

**Authors:** Qingzhu Wang, Wanying Zhang, Josephine Chen, Kate T. Stenson, Shikha Parsai, Prerana Bangalore Parthasarathy, Chia-Feng Liu, Thomas Garrett, Jung Lin, Anlin Hou, Riya A. Shimpi, Eric Gu, Mei Yin, Logan Shannon, Sadie Johnson, Belinda B. Willard, E. Rene Rodriguez, Charlie Androjna, Rohit Moudgil, Yilun Sun, Jennifer S. Yu, Christopher Nguyen, Tyler J. Alban, Timothy A. Chan, Sathyamangla V. Naga Prasad, W.H. Wilson Tang, Jianhong Lin, Jianjun Zhao

**Affiliations:** 1 Department of Cancer Sciences, Cleveland Clinic, Cleveland, Ohio.; 2 Department of Cardiovascular and Metabolic Sciences, Cleveland Clinic, Cleveland, Ohio.; 3 Department of Biomedical Sciences, College of Medicine, Northeast Ohio Medical University, Rootstown, Ohio.; 4 Cardiovascular Innovations Research Center, Cleveland Clinic, Cleveland, Ohio.; 5 Image Core, Cleveland Clinic, Cleveland, Ohio.; 6 SAIC, Cleveland Clinic, Cleveland, Ohio.; 7 Proteomics Core, Cleveland Clinic, Cleveland, Ohio.; 8 Department of Laboratory Medicine, Robert J. Tomsich Pathology & Laboratory Medicine Institute, Cleveland Clinic, Cleveland, Ohio.; 9 Department of Cardiovascular Medicine, Heart Vascular and Thoracic Institute, Cleveland Clinic, Cleveland, Ohio.; 10 Department of Pharmacology, University of Maryland School of Medicine, Baltimore, Maryland.; 11 Department of Radiation Oncology, Neurological Institute, Cleveland Clinic, Cleveland, Ohio.

## Abstract

Anthracyclines are potent chemotherapeutic agents known for their efficacy in treating various cancers via inhibition of topoisomerase II α (TOP2A). However, their clinical use is limited due to cardiotoxicity, primarily attributed to off-target inhibition of topoisomerase II β (TOP2B) in cardiomyocytes. The well-accepted mechanism involves TOP2B inhibition as a key driver of this toxicity. In this study, we identify a novel mechanism of anthracycline-induced cardiotoxicity (AIC) involving upregulated TOP2B expression and its direct impact on cardiomyocyte function. Our data show that doxorubicin significantly increased TOP2B protein levels in cardiomyocytes in AIC mouse models. The cardiomyocyte-specific, tamoxifen-inducible *TOP2B* transgenic mice exhibited pathophysiologic features consistent with doxorubicin-induced cardiotoxicity, even without exposure to anthracyclines. Additionally, we discovered that TOP2B binds to SMYD1, a histone methyltransferase critical for muscle cell function. Mutations in *SMYD1* are known to cause cardiomyopathy and heart failure in humans, and loss of *Smyd1* in mice results in a phenotype resembling AIC. More importantly, TOP2B antisense oligonucleotide (ASO) pretreatment can succussfully prevent AIC in *TOP2B* transgenic mice and AIC mouse models. Our findings reveal a novel role for TOP2B in AIC, demonstrating that its upregulation disrupts SMYD1 function in cardiomyocytes, contributing to cardiotoxicity. This study also highlights the therapeutic potential of targeting TOP2B using ASO for preventing AIC in patients with cancer, offering new insights into cardioprotective strategies.

**Significance:**

Anthracycline chemotherapy can cause severe and sometimes fatal heart damage, limiting its clinical use. We identify TOP2B upregulation as a key driver of cardiotoxicity and demonstrate that ASO therapy targeting TOP2B prevents heart failure and improves survival in preclinical models, providing a promising strategy to protect patients with cancer during chemotherapy.

## Introduction

Anthracycline-induced cardiotoxicity (AIC) is a major clinical problem, but its underlying mechanism is mostly unclear (1). Anthracyclines, which are first-line chemotherapeutic agents for a wide variety of common cancers (e.g., breast cancer, sarcomas, lymphomas, and leukemias), have toxic effects on the cardiovascular system, including cardiac arrhythmias, hemodynamic instability, inflammatory cardiomyopathy, and congestive heart failure (2). The mechanism of action of anthracyclines is widely thought to be dependent on the inhibition of topoisomerase enzymes (3), which have central roles in DNA replication, transcription, chromosome segregation, and recombination. Of the two topoisomerase 2 isozymes, topoisomerase II α (TOP2A) is highly expressed in cancer cells and is required for cell division (4). However, adult cardiomyocytes express only topoisomerase II β (TOP2B), which is involved in DNA transcription, but not DNA replication, with unique roles in chromosome structure (4). Interestingly, topoisomerases have recently been suggested to be particularly important in the transcription of long genes, suggesting that topoisomerase function may be uniquely important in certain cell types (5). Published data show that TOP2A mediates doxorubicin’s tumoricidal activity, whereas TOP2B mediates doxorubicin’s cardiotoxicity (6). However, TOP2B is expressed broadly in other tissues such as kidneys to a similar level as in the heart (7). It thus remains unclear how TOP2B-dependent toxicity (production of anthracycline-bound DNA–TOP2B complexes) leads to the prominent and organ-specific cardiotoxicity experienced in patients and may not be involved in other tissue, hampering the development of effective therapeutics for AIC.

At present, dexrazoxane is currently the only FDA-approved drug specifically indicated for preventing AIC. Its cardioprotective effects stem from multiple mechanisms, primarily by inhibiting TOP2B activity in cardiomyocytes—preventing the formation of harmful DNA cleavage complexes caused by anthracyclines—and by promoting TOP2B degradation via the proteasome. Additionally, as an EDTA derivative, it chelates iron, reducing the formation of reactive oxygen species (ROS) from iron–anthracycline complexes. Together, these actions protect the heart from both DNA damage and oxidative stress during anthracycline chemotherapy. Dexrazoxane is FDA-approved only for patients with breast cancer who have already received 300 mg/m^2^ of anthracyclines. Although dexrazoxane reduces oxidative stress by iron chelation, its interference with TOP2A catalytic activity raised concern regarding potential impact on antitumor efficacy due to its off-target effect on degradation of TOP2A in tumor cells and its role on reducing the formation of ROS. Also, dexrazoxane is not routinely used in practice, and prescription to children is currently off label in the United States (8). In 2011, its use in children was contraindicated by the European Medicines Agency over concerns of increased risk of infection, myelosuppression, and second primary malignancies and because its efficacy in children had not then been established (9). The previous contraindication for pediatric patients has been removed from the European label following evidence of safety and cardio protection. Nevertheless, a new-potency TOP2B-targeted therapy with better specificity is needed to prevent cardiotoxicity.

Using AIC mouse models and *TOP2B* transgenic mouse models, we have found that high expression of TOP2B itself is the direct mediator of AIC, without TOP2B–anthracycline–DNA complex formation in cardiomyocytes. Our studies have revealed that first, doxorubicin increased TOP2B protein level in response to DNA damage in cardiomyocytes. Second, the cardiomyocyte-specific and tamoxifen-inducible *TOP2B* transgenic (α*-MHC-MerCreMer*^*+/−*^*TOP2B*^*LSL/−*^) mouse recapitulated the pathophysiologic features of AIC without doxorubicin treatment. Third, cardiac magnetic resonance imaging (MRI) and echography determined both acute heart failure and chronic heart failure in two different *TOP2B* transgenic mouse models with different dose of tamoxifen treatment protocols. Fourth, spatial transcription analysis verified heart failure signature related to SMYD1 gene downstream genes developed in TOP2B transgenic mice. Fifth, highly expressed TOP2B binds cardiomyocyte-specific protein SMYD1, inducing dysregulation of downstream genes, which may explain the tissue-specific toxicity observed in the heart. SMYD1 is a muscle-specific histone methyltransferase and plays a pivotal role in normal cardiac muscle growth and differentiation (10), as well as dilated cardiomyopathy in humans (11). Conditional cardiomyocyte-specific *SMYD1* knockout (KO) mice produce a myopathy characterized by increased internal nuclei, hypotrophy, myofibrillar disarray, and weakness (12). Finally, TOP2B antisense oligonucleotide (ASO) treatment prolong the survival of both the TOP2B transgenic (α*-MHC-MerCreMer*^*+/−*^, *TOP2B*^*LSL/−*^) mouse model and AIC model. The superior preventive efficacy of TOP2b ASO compared with dexrazoxane in those mouse models highlights its potential as a promising therapeutic strategy for AIC in patients with cancer.

## Materials and Methods

### Mice

Doxorubicin was administered intravenously at a dose of 5 mg/kg once per week for four consecutive weeks to induce cardiotoxicity in the AIC model. Heart tissues were collected 7 days after the final dose for Western blot and quantitative PCR (qPCR) analysis. A CRISPR-mediated recombination at the Rosa26 3′UTR locus was used to generate human TOP2b conditional transgenic mice termed *hTOP2B*^*LSL/−*^ mice. *hTOP2B*^*LSL/−*^ mice were generated by inserting a CAG promoter–driven plasmid with 5X STOP cassette flanked by LoxP sites (LSL) at the start of the *hTOP2B cDNA* at the mouse *Rosa26 3*′*UTR* locus. The STOP cassette prevents expression of the *hTOP2B* gene. To remove the STOP cassette and activate hTOP2B expression in cardiomyocytes *in vivo*, the *hTOP2b*^*LSL*^ mice were mated with α*-MHC-MerCreMer*^*+/−*^ mice (The Jackson Laboratory), in which the ERT moiety retains Cre recombinase activity in the cytoplasm of most of the cardiomyocytes until tamoxifen administration, which releases this block and promotes recombination of genomic LoxP sites. Six-week-old α*-MHC-MerCreMer*^*+/−*^*hTOP2b*^*LSL/−*^ mice received intraperitoneal injections of one dose of 80 mg/kg of tamoxifen to generate acute heart failure mouse model. To more recapitulate multiple dose of doxorubicin treatment in the clinic, 16-week-old α*-MHC-MerCreMer*^*+/−*^*hTOP2b*^*LSL/−*^ mice received intraperitoneal injections of 1.5 mg/kg/week of tamoxifen for consecutive 8 weeks to generate chronic heart failure mouse model. Same-age α*-MHC-MerCreMer*^*+/−*^*sibling* mice with same doses of tamoxifen injection were used as control.

### MRI

To assess changes in the cardiac function, MRI imaging was performed on a cohort of control and transgenic mice. Data sets were acquired on a 7T MRI scanner (Biospec 70/20, Bruker), utilizing a four-channel brain array surface coil for cardiac imaging. The mice were anesthetized with 2% isoflurane and positioned within a custom-designed 3D printed adapter (Bruker). The adapter allowed for the inverted placement of the coil on the MRI bed, thereby ensuring that the heart was positioned as close as possible to the array coil element. Mice were placed in a supine position with their forelimbs extended rostrally to either side of their thorax and hind limbs extended caudally. Images were obtained using a standard short-axis self-gated FLASH sequence, with a range of 8 to 10 slices, covering the heart from the base to the apex. Self-gating was performed using an in-slice navigator echo. The sequence parameters were as follows: TE = 4 milliseconds, TR = 11.77 milliseconds, flip angle = 10 degrees, field of view = 25 × 17 mm^2^, acquisition matrix (NF × NP) = 250 × 170, and slice thickness = 0.8 mm. Cine movies with 30 frames per slice were reconstructed retrospectively based on an average respiration rate of 35 BPM. Respiratory rate, heart rate, and temperature were monitored throughout the image acquisition. The MRI data acquired allowed for quantification of left ventricular (LV) and right ventricular (RV) sizes, anterior and posterior wall thicknesses, and LV and RV cavity dilations.

### Echocardiography

Cardiac function was assessed by transthoracic echocardiography using a high-frequency ultrasound system (Vevo 2100, VisualSonics) equipped with a 30 MHz transducer. Mice were anesthetized with 1% to 2% isoflurane. Parasternal long-axis and short-axis views were obtained to evaluate LV function. LV ejection fraction and fractional shortening were calculated from M-mode tracings at the mid-papillary level using standard formulas. All measurements were averaged from at least three consecutive cardiac cycles and analyzed in a blinded manner.

### Human heart tissue preparation

Explanted human heart specimens were obtained from nonfailing heart donors and patients with AIC at the time of heart transplantation in accordance with Cleveland Clinic Foundation Institutional Review Board approval.

### Immunoprecipitation of hTOP2b-binding proteins

hTOP2B antibody (Santa Cruz Biotechnology, sc365071) was then used to pull down TOP2B-binding proteins from heart tissue lysate of α*-MHC-MerCreMer*^*+/−*^*TOP2B*^*LSL/−*^ mice 3 days after intraperitoneal injections of 80 mg/kg of tamoxifen. Normal mouse IgG (Santa Cruz Biotechnology, sc2025) was used as control. Pulldown samples were run on SDS-PAGE gels. The bands from the gel were cut out, washed/destained in 50% ethanol containing 5% acetic acid, dehydrated in acetonitrile, reduced with dithiothreitol, and alkylated with iodoacetamide prior to digestion. All bands were completely digested in-gel by using trypsin 5 μL (10 ng/μL) in 50 mmol/L ammonium bicarbonate overnight at room temperature. Peptides were extracted from the polyacrylamide in two aliquots of 30 μL 50% acetonitrile containing 5% formic acid. The extracts were combined and evaporated to <10 μL in a SpeedVac and then resuspended in 1% acetic acid to make up a final volume of ∼30 μL for liquid chromatography–mass spectrometry (LC-MS) analysis.

### MS

The LC-MS system was an LTQ-Obitrap Elite hybrid mass spectrometer system (Thermo Fisher Scientific) and a Dionex 15 cm × 75 μm id Acclaim Pepmap C18, 2 μm, 100 Å reversed-phase capillary chromatography column. Extracts were injected in 5 μL volumes, and the peptides were eluted with an acetonitrile/0.1% formic acid gradient at a flow rate of 0.25 μL/minute introduced into the mass spectrometer source. The microelectrospray ion source was operated at 2.5 kV. The digest was analyzed using the data-dependent multitask capability of the instrument, acquiring full-scan mass spectra in the Orbitrap at a resolution of 60,000 to determine peptide molecular weights and production spectra in the ion trap to enable determination of the amino acid sequence in sequential scans. Data were analyzed by using all the collected CID spectra and searching the NCBI human reference sequence database (March 2015 with 99,739 entries) with the search programs Mascot (version 2.3.0) and SEQUEST (version 2.2). The data were uploaded into Scaffold (version 4.0) for protein and peptide validation. To identify proteins, a threshold of at least five CID spectra (spectral counts) was set; the proteins identified in TOP2B pulldown/control pulldown samples at a level greater than 2.5-fold were collected by filtration and marked as TOP2B-binding proteins for further analysis.

### Immunohistochemistry

Formalin-fixed, paraffin-embedded (FFPE) tissue sections were deparaffinized and then incubated with rabbit anti-TOP2B polyclonal antibody (Novus, NB100-40842) and rabbit anti-SMYD1 antibody (Santa Cruz Biotechnology, Inc., sc514804) at 4°C overnight. After incubation with horseradish peroxidase (HRP)-conjugated goat anti-rabbit secondary antibody, the signal was detected using a DAB Substrate kit (Abcam, ab64238) according to the manufacturer’s instructions. Images were obtained using a phase-contrast microscope (Leica DM2000 LED) equipped with a digital camera (Leica DMC 2900).

### Immunoblotting

Total cellular protein samples were isolated by RIPA buffer with protease and phosphatase inhibitors (Thermo Fisher Scientific). Where indicated, mitochondrial and cytosolic proteins were isolated using Mitochondria Isolation Kit (Thermo Fisher Scientific), per the manufacturer’s instructions. Proteins were resolved on 4% to 12% Bis-Tris polyacrylamide gels (Thermo Fisher Scientific) and transferred onto polyvinylidene difluoride membranes (Millipore). Membranes were blocked with 5% nonfat milk and incubated overnight with rabbit anti-TOP2B polyclonal antibody (Novus, NB100-40842), hTOP2B antibody (Santa Cruz Biotechnology, Inc., sc365071), mouse anti SMYD1 (Santa Cruz Biotechnology, Inc., sc514804), mouse anti-TITIN (Santa Cruz Biotechnology, Inc., sc271945), and rabbit anti–enolase 3 (ENO3; GTX113429) overnight at 4°C. Membranes were then washed and incubated with HRP-linked anti-rabbit IgG secondary (Cell Signaling Technology, #7074) or HRP-linked anti-mouse IgG secondary (Cell Signaling Technology, #7076S). Detection of chemiluminescence was carried out using a SuperSignal West Femto Maximum Sensitivity Substrate kit (Thermo Fisher Scientific).

### Immunofluorescence microscopy

Fresh-frozen mouse heart tissues were embedded in optimal cutting temperature compound and sectioned at 5 μm thickness using a cryostat microtome. Tissue sections were air-dried, washed in phosphate-buffered saline (PBS), and fixed in prechilled acetone (−20°C). Sections were then permeabilized with 0.1% Triton X-100 in PBS. After blocking, slides were incubated overnight at 4°C with the following primary antibodies: human TOP2B (hTOP2B; Santa Cruz Biotechnology, sc-365071) and cardiac troponin I (cTnI; Sino Biological, 100758-T08). Following primary antibody incubation, sections were washed with PBS and incubated with appropriate fluorescent secondary antibodies for 1 hour at room temperature: goat anti-rabbit Alexa Fluor 488 (Thermo Fisher Scientific, cat. #A-11008, RRID: AB_143165) and goat anti-mouse Alexa Fluor 594 (Thermo Fisher Scientific, cat. #A-11005, RRID: AB_2534073). After washing, slides were mounted with antifade DAPI-containing mounting medium. Fluorescent images were acquired using a Leica TCS SP8 confocal microscope. Image analysis was performed using Leica LAS X software. For selected samples, z-stack images were obtained at sequential optical sections and processed to generate maximum intensity projections (MIP). Brightness and contrast adjustments were applied uniformly to images acquired under identical settings for visualization purposes.

### hTOP2b and SMYD1 binding site identification

To further elucidate the binding site of SMYD1 with TOP2B, a series of eGFP-SMYD1 plasmids were generated. These plasmids included the full-length human SMYD1 sequence as well as various truncated fragments of human SMYD1. The plasmid constructs were designed and synthesized to retain the appropriate coding regions while enabling the identification of specific interaction domains. The 3FLAG-hTOP2B plasmid was constructed by using hTOP2B cDNA cloned into empty vectors. Each construct was verified by sequencing to confirm accuracy and integrity before use in subsequent experiments. Human immortalized cardiomyocyte AC16 cells were transfected with 3FLAG-hTOP2B plasmid and individual eGFP-SMYD1 plasmids. Flag tag antibody was then used to pull down individual eGFP-SMYD1 proteins. Anti-IgG was used as control. Pulldown samples were run on SDS-PAGE gels and for Western blot analysis of FLAG and eGFP. AC16 cells were also seeded in six-well plates on sterile glass coverslips for immunofluorescence microscopy study. The samples were washed, fixed with −20°C acetone, permeabilized with 0.1% Triton X-100, and then incubated with primary antibodies: mouse anti-FLAG antibody (Sigma cat. #SLCQ9255, RRID: AB_259529), washed with 1× PBS. Then the tissues were stained with the secondary antibody goat anti-mouse (Abcam cat. #ab150115, RRID: AB_2687948). Images were captured by confocal microscopy (Leica TCS SP8). Images were analyzed with Leica LAS-X software. In some instances, a set of images were taken at varying z-stack depth and compressed to create a MIP. Brightness and contrast were uniformly altered in images taken with identical settings for visualization.

### Transmission electron microscopy analysis

Mouse hearts were submerged in EM grade 2.5% glutaraldehyde and 4% paraformaldehyde in 0.2 mol/L sodium cacodylate buffer (pH 7.4) at 4°C immediately after collection and fixed at 4°C overnight. After washing three times for 5 minutes in sodium cacodylate buffer (0.2 mol/L, pH 7.3), the heart tissues were fixed in 1% aqueous osmium tetroxide for 60 minutes at 4°C, then washed twice for 5 minutes with sodium cacodylate buffer, and rinsed once with maleate buffer (pH 5.1, 5 minutes). After changing to 1% uranyl acetate in maleate buffer, the cochlear tissues were stained for 60 minutes; the uranyl acetate was removed. The samples were then washed three times for 5 minutes with maleate buffer and then dehydrated with ascending grades of ethanol and finally embedded in Epon resin (Electron Microscopy Science). Ultrathin sections (85 nm) were cut by means of an EM UC7 ultramicrotome (Leica Microsystems), then successively stained with uranyl acetate and lead citrate, and examined with a TEM instrument at 80 kV (Tecnai G2 SpiritBT, FEI).

### Visium spatial single-cell RNA sequencing

The histology workflow utilized the Visium CytAssist Spatial Gene Expression for FFPE (following protocol CG000520). Tissue sections, prepared according to the Visium CytAssist Spatial Gene Expression for FFPE – Tissue Preparation Guide (protocol CG000518), were cut into 5-μm slices and mounted on Fisherbrand Plus Microscope Slide. These sections were hematoxylin and eosin–stained after deparaffinization and then imaged and decoverslipped, followed by hematoxylin destaining and decrosslinking (Protocol CG000520). Using the Visium CytAssist instrument, analytes from the tissue sections were transferred to a Visium CytAssist Spatial Gene Expression slide with 6.5 × 6.5 mm capture area. The subsequent probe extension and library construction steps followed the standard Visium for FFPE workflow outside of the instrument. Sequencing libraries, created with paired-end dual-indexing (28 cycles read 1, 10 cycles i7, 10 cycles i5, and 50 cycles read 2S). Sequencing libraries were demultiplexed with NovaSeq (Illumina). Differential gene expression between α*-MHC-MerCreMer*^*+/−*^ mice and α*-MHC-MerCreMer*^*+/−*^*hTOP2b*^*LSL/−*^ mice cardiomyocyte populations was performed in Seurat (R). After standard preprocessing (normalization, scaling, and clustering) and identification of the relevant cardiomyocyte cluster(s), marker genes were identified using Seurat FindMarkers with the Wilcoxon rank-sum test. Genes were tested across groups and reported as average log_2_ fold change (avg_log_2_FC) with associated *P* values. Multiple testing correction was performed using the Benjamini–Hochberg method to obtain adjusted *P* values (*P*_val_adj). Genes with adjusted *P* < 0.05 were considered differentially expressed. For downstream enrichment, DEGs were separated into upregulated (avg_log_2_FC > 0) and downregulated (avg_log_2_FC < 0) sets. Gene Ontology (GO) enrichment analysis was performed on upregulated and downregulated DEG lists using clusterProfiler (R). Gene symbols were converted to Entrez IDs using the appropriate organism annotation database. Overrepresentation analysis was conducted using enrichGO with ont = “BP” (Biological Process). The background gene universe was defined as all genes detected in the analyzed cardiomyocyte population (or all genes tested in FindMarkers). Enriched GO terms were considered significant at FDR-adjusted *P* < 0.05 (Benjamini–Hochberg). The top enriched BP terms (ranked by FDR or gene ratio) were visualized as a horizontal bar plot, with upregulation-associated terms shown in red and downregulation-associated terms shown in blue. Data are publicly available on GEO (GSE319842).

### Design and transfection of TOP2B ASO

To target human TOP2B, we designed and synthesized 30 Gampers ASOs (Supplementary Table S1; Eurofins Genomics). Each ASO was carefully optimized for specificity to the TOP2B mRNA sequence. First, sequence-dependent off-target hybridization risk was evaluated using NCBI BLAST against the mouse and human transcriptome to identify potential partial complementarity outside the intended target. No significant unintended full-length complementarity was identified, and no high-affinity off-target transcripts with ≤2 mismatches were detected. Second, the ASO sequence was analyzed for immunostimulatory CpG motifs known to activate TLR9-mediated innate immune responses. The sequence does not contain canonical CpG dinucleotide motifs associated with strong immune activation. Third, we evaluated GC content, melting temperature, and predicted secondary structure to ensure appropriate hybridization specificity and avoid self-complementary hairpin formation that could alter biodistribution or toxicity. AC16 human cardiomyocyte cells were cultured in standard growth media under optimal conditions. Cells were transfected with 20 μg/mL of the TOP2B-targeting ASOs using Lipofectamine RNAiMAX (Thermo Fisher Scientific) according to the manufacturer’s protocol. After 48 hours of transfection, cell lysates were collected, and TOP2B protein levels were determined via Western blot analysis. Western blotting was performed using rabbit anti-TOP2B polyclonal antibody (Novus, NB100-40842) to confirm the knockdown efficiency of the ASOs.

### AC16 cell differentiation and metabolic conditioning

AC16 human cardiomyocytes (Millipore, SCC109) were used at passages 4 to 8; all were authenticated by short tandem repeat profiling and tested negative for *Mycoplasma*. AC16 human cardiomyocyte cells were subjected to a differentiation and metabolic conditioning protocol designed to promote a shift from a glycolytic metabolism toward oxidative phosphorylation. Cells were first expanded in standard growth medium consisting of DMEM/F12 supplemented with 10% fetal bovine serum (FBS) and 1% penicillin/streptomycin (P/S) and cultured to approximately 80% to 90% confluence. To initiate differentiation and metabolic reprogramming, the growth medium was replaced with a differentiation medium composed of DMEM/F12 supplemented with 2% horse serum, 1× insulin–transferrin–selenium, 1% FBS, and 0.5% P/S. This medium composition is known to support cardiomyocyte maturation and enhance mitochondrial biogenesis and function. Cells were maintained in differentiation medium for 14 days, with medium changes every 2 to 3 days. No passaging or enzymatic dissociation (e.g., trypsinization) was performed during this period to preserve cell–cell contacts and promote stable differentiation. Successful differentiation was confirmed by the upregulation of SMYD1, a cardiac-specific histone methyltransferase and differentiation marker, as assessed by Western blotting. Increased SMYD1 expression is associated with enhanced mitochondrial function and oxidative metabolism, consistent with the expected shift in AC16 cell metabolic phenotype. Under these conditions, AC16 cells progressively adopt a more oxidative metabolic phenotype, characterized by increased reliance on mitochondrial respiration.

### Oxygen consumption assay

The functional activity of differentiated AC16 cells mitochondria was measured by using a Seahorse XFe24 Analyzer and a Seahorse XF Cell Mito Stress Test Assay (Agilent Technologies). The oxygen consumption rate (OCR) of cells was measured according to manufacturer’s instructions. A measure of 100 nmol/L TOP2B ASO (TOP2B ASO 18) or 100 nmol/L GFP ASO were transfected 24 hours prior to the experiment and then resuspended, and 5 × 10^4^ cells were seeded into 24-well plates and allowed to attach overnight, according to the manufacturer’s instructions, while being treated with doxorubicin at 1 μg/mL for 24 hours. The culture medium was removed and replaced with dye and buffer-free XF DMEM medium (Agilent Technologies). Then cells were subjected to OCR detection in the presence of oligomycin (1 μmol/L), carbonyl cyanide p-trifluoromethoxy phenylhydrazone (FCCP, 2 μmol/L), and a combination of rotenone (0.5 μmol/L) and antimycin A (0.5 μmol/L). Basal OCR = OCR before the injection of oligomycin. Adenosine triphosphate (ATP) synthesis–linked OCR (ATP-linked) = basal OCR − OCR following oligomycin injection. Maximum OCR = OCR following the injection of FCCP. Reserve = maximum respiration − basal OCR. Proton leak–linked OCR = uncoupled OCR following oligomycin − non-mitochondrial OCR following injection of rotenone and antimycin A. Nonmitochondrial OCR = OCR following the injection of rotenone and antimycin A. Significant outliers were removed prior to analysis.

### Evaluating the efficacy of ASO in preventing cardiomyopathy *in vivo*

To evaluate the efficacy of ASO therapy in preventing cardiomyopathy, we utilized a tamoxifen-inducible, cardiomyocyte-specific TOP2B transgenic mouse model (α*-MHC-MerCreMer*^*+/−*^*hTOP2B*^*LSL/−*^) as well as an AIC model in wild-type C57BL/6J mice. For the TOP2B transgenic cardiomyopathy model, transgenic TOP2B expression was induced by a single intraperitoneal injection of tamoxifen (80 mg/kg). Mice received either TOP2B ASO-18 (10 mg/kg, subcutaneously) or dexrazoxane (100 mg/kg, intraperitoneally) 30 minutes before tamoxifen injection, whereas control mice received saline (intravenously). The day of tamoxifen administration was defined as day 1 for survival analysis. For the AIC model, doxorubicin was administered at 5 mg/kg intravenously once per week for four consecutive weeks to induce cardiotoxicity in wild-type C57BL/6J mice. Mice received either TOP2B ASO-18 (2.5 mg/kg, subcutaneously) or dexrazoxane (25 mg/kg, intraperitoneally) 30 minutes prior to each doxorubicin injection. Control mice received saline injections. The day following the final doxorubicin dose was defined as day 1 for survival analysis. Survival curves were generated using the Kaplan–Meier method, and group comparisons were performed using Cox proportional hazards regression analysis. Statistical analyses were conducted using GraphPad Prism, with *P* < 0.05 considered statistically significant.

### Statistical analysis

A two-tailed Student *t* test was performed using SPSS (version 17.0) to compare independent pairs of groups. *P* ≤ 0.05 was statistically significant.

## Results

### TOP2B expression in heart sample in patients with AIC

**Figure 1.**
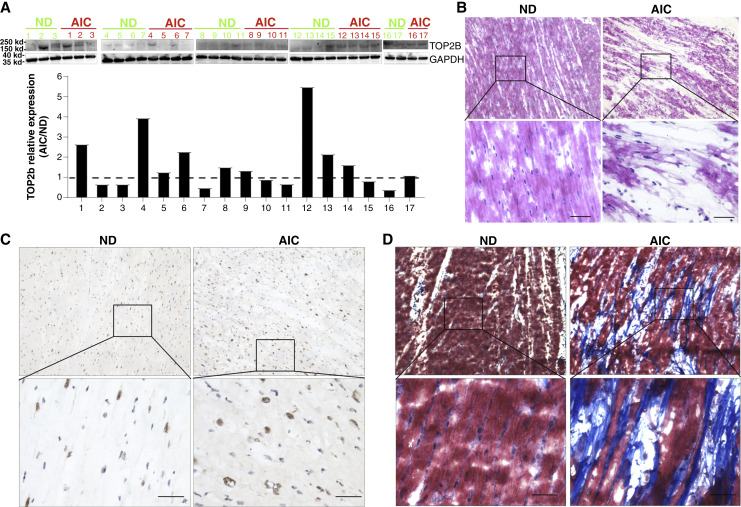
Doxorubicin induces TOP2B overexpression in human AIC specimens. **A,** Western blot analysis showing the expression levels of TOP2B and GAPDH in heart tissue samples from ND patients and patients with AIC. **B,** Hematoxylin and eosin stains of heart tissue sections from ND patients and patients with AIC, illustrating tissue morphology and cellular integrity. **C,** IHC staining showing the localization and expression of TOP2B in heart tissue sections from ND patients and patients with AIC. **D,** Masson’s Trichrome staining of heart tissue sections from ND patients and patients with AIC, highlighting fibrosis in purple color in the tissue.

Published data had showed that TOP2A mediates doxorubicin’s tumoricidal activity, whereas inhibition of TOP2B mediates doxorubicin’s cardiotoxicity (6). We had collected the AIC patient samples from patients with end-stage heart failure undergoing cardiac transplantation (Supplementary Table S1). In heart specimens from patients with AIC, higher expression of TOP2B is observed in heart samples of 10 of the 17 patients with AIC enrolled in the study compared with 17 age- and sex-matched heart samples from normal donors (ND; Fig. 1A). Cardiomyocyte histology of the patients with AIC disorganized myofibrils compared with ND patients (Fig. 1B), with higher TOP2b expression (Fig. 1C) and collagen deposition (Fig. 1D). These findings contradict the prevailing theory that inhibition of TOP2B by anthracycline drugs is the primary driver of AIC. However, it is important to note that the AIC patient samples analyzed were obtained from individuals with end-stage heart failure undergoing cardiac transplantation, which does not fully represent the earlier stages of TOP2B dysregulation following doxorubicin treatment. Consequently, using only end-stage patient samples makes it difficult to delineate how TOP2B contributes to the development of functional cardiac defects without complementary *in vivo* animal studies.

### TOP2B upregulation in response to doxorubicin induces DNA damage

**Figure 2.**
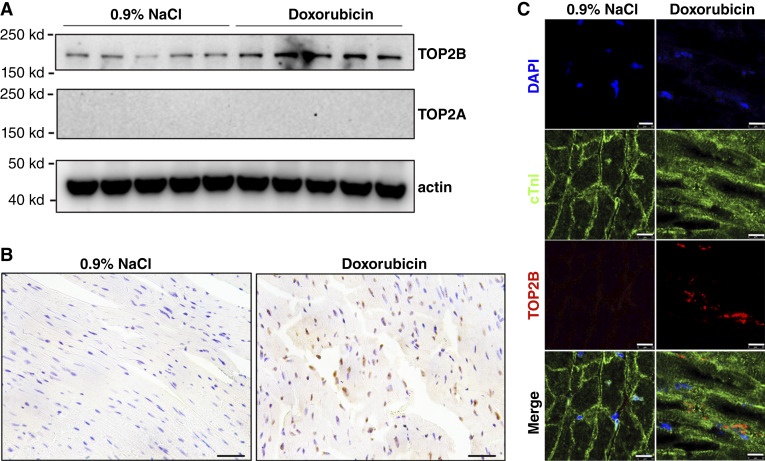
Doxorubicin induces TOP2B overexpression in the AIC mouse model. **A,** Western blot analysis showing increased expression of TOP2B protein in the AIC mouse model treated with doxorubicin (5 mg/kg/week, i.v., for four consecutive weeks) in mice with AIC. **B,** IHC staining confirming TOP2B overexpression in tissue samples from the same model. **C,** Immunofluorescence staining of cardiac sections demonstrating increased nuclear TOP2B (red) in cardiomyocytes following doxorubicin treatment. cTnI (green) marks cardiomyocytes, and DAPI (blue) labels nuclei. Merged images show enhanced nuclear localization of TOP2B in cardiomyocytes of doxorubicin-treated hearts relative to controls. Scale bars as indicated.

In order to examine the TOP2B changes after anthracycline drug treatment, we used AIC mouse model wherein treatment with doxorubicin (5 mg/kg/week) for four consecutive weeks for AIC mouse model as described in the literature (13). This recapitulating histologic changes in human AIC. Analysis of TOP2B expression in doxorubicin mouse models after 1 week of the last dose revealed that TOP2B protein is upregulated in cardiomyocytes (Fig. 2A) but not significantly in mRNA level (Supplementary Fig. S1). TOP2B protein level had been found also upregulated in AIC mouse models by using immunohistochemistry (IHC) methods (Fig. 2B; Supplementary Fig. S2). To further define the cellular localization of TOP2B upregulation, we performed immunofluorescence staining of cardiac sections using antibodies against TOP2B and cardiac troponin I (cTnI), a structural marker of cardiomyocytes. In saline-treated controls, TOP2B expression was low and diffusely distributed, and cTnI exhibited the expected organized sarcomeric pattern. In contrast, doxorubicin treatment resulted in marked nuclear accumulation of TOP2B within cTnI-positive cardiomyocytes (Fig. 2C). Notably, cTnI staining appeared less organized and more diffuse in doxorubicin-treated hearts, suggesting disruption of sarcomere structure consistent with cardiomyocyte injury. These findings indicate that TOP2B upregulation occurs specifically in cardiomyocytes following anthracycline exposure and may be associated with structural alterations characteristic of cardiotoxicity.

### TOP2B transgenic mice develop cardiomyocyte damage after TOP2B is induced

**Figure 3.**
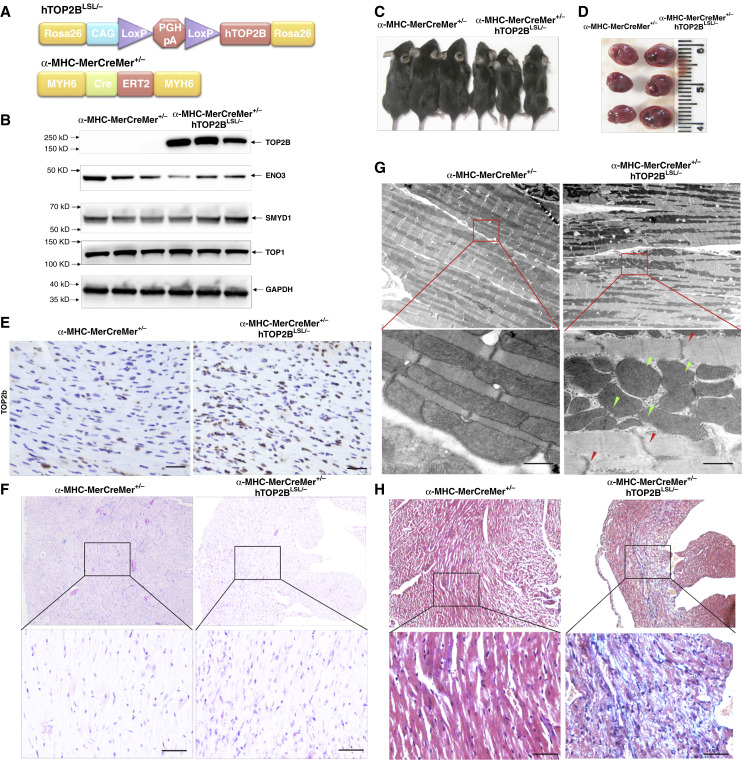
TOP2B conditional transgenic mouse model. **A,** Schematic diagram of α-MHC-MerCreMer^+/−^, TOP2B^LSL/−^ conditional transgenic mouse model. **B,** Western blot result of expression in heart tissue from α-MHC-MerCreMer^+/−^, TOP2B^LSL/−^ transgenic mice compared with α-MHC-MerCreMer^+/−^ control mice. Representative image of (**C**) mice and (**D**) heart in α-MHC-MerCreMer^+/−^, TOP2B^LSL/−^ transgenic mice (*n* = 3) and α-MHC-MerCreMer^+/−^ control mice (*n* = 3) after tamoxifen injection 3 days. Hematoxylin and eosin staining (**E**), IHC of TOP2b (**F**) and EM (**G**) checking (**H**) Masson’s Trichrome staining of heart tissue sections from α-MHC-MerCreMer^+/−^, TOP2B^LSL/−^ transgenic mice (*n* = 3) and α-MHC-MerCreMer^+/−^ control mice after tamoxifen treatment at 6 weeks of age.

To determine whether AIC develops in mice via anthracycline mediated TOP2B overexpression, we generated a new Rosa 26 TOP2B conditional transgenic mouse model (Fig. 3A). To mimic the effect of doxorubicin-induced heart TOP2B overexpression, we crossed *Rosa 26 TOP2B*^*LSL/−*^ mice with α*-MHC-MerCreMer-CreERT2*^*+/−*^ mice, in which Cre-induced expression of TOP2B is controlled by the CAG promoter, a well-established strategy for generating transgenic mice that ensures high expression in organs after tamoxifen administration (14). In 6-week-old mice, after given one 80 mg/kg dose of tamoxifen, dissection revealed high TOP2B expression in hearts (Fig. 3B). The cardiomyocyte-specific TOP2B transgenic (α*-MHC-MerCreMer*^*+/−*^*TOP2B*^*LSL/−*^) mice developed growth retardation (Fig. 3C) and heart dilatation (Fig. 3D) after TOP2b was induced. Histology checking verified TOP2B overexpression in cardiomyocyte (Fig. 3E), cell nuclear aggregation (Fig. 3F) and mitophagy [Fig. 3G (green arrow)], abnormal myofibrillar architecture [Fig. 3G (red arrow)], and collagen deposition (Fig. 3H).

### TOP2B transgenic mice have much shorter survival due to acute heart failure

**Figure 4.**
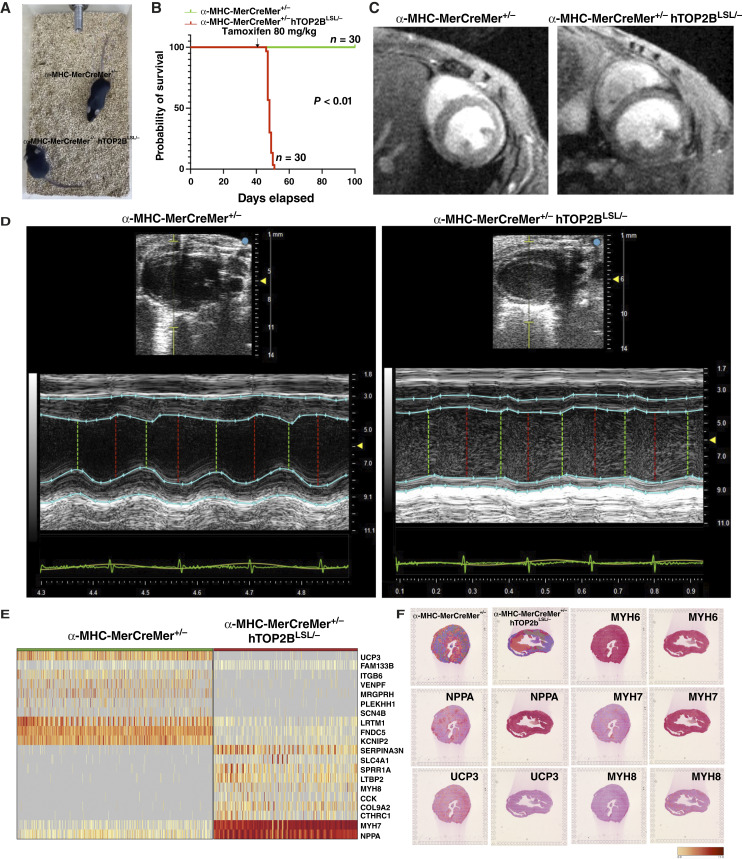
TOP2B conditional transgenic mouse model developed acute heart failure. **A,** Snapshot from a video showing the movement of α-MHC-MerCreMer^+/−^, TOP2B^LSL/−^ transgenic mice, which developed heart failure-related phenotypes including weight loss, slow movement, and dyspnea. **B,** Kaplan–Meier survival curves comparing the survival rates of control and TOP2B transgenic mice. **C,** MRI images showing the heart at the basal levels in control and transgenic mice. **D,** Graphs from echography showing the ejection fraction (EF) and cardiac output (CO) in control and TOP2B transgenic mice. **E,** List of dysregulated genes identified through 10× spatial transcriptome analysis. **F,** Image showing the spatial distribution of deregulated genes including NPPA, UCP3, MYH6, MYH7, and MYH8 in the heart tissue of transgenic mice.

After a standard 80 mg/kg single dose of tamoxifen injection from days 7 to 10, all TOP2B transgenic (α*-MHC-MerCreMer*^*+/−*^*TOP2B*^*LSL/−*^) mice developed acute heart failure phenotype, including slow movement, difficulty in breathing, and weakness (Fig. 4A; Supplementary Video S1), and then quickly died from acute heart failure (Fig. 4B), but none of the α*-MHC-MerCreMer*^*+/−*^ control mice exhibited these changes or died. There were obvious morphology changes in the thickness of the left ventricle (LV; Fig. 4C; Supplementary Videos S2 and S3) and significantly reduced EF and CO in TOP2B transgenic (α*-MHC-MerCreMer*^*+/−*^*TOP2B*^*LSL/−*^) mice in echocardiograph (Fig. 4D; Supplementary Table S2). To define the transcriptional changes induced by TOP2B activation in cardiomyocytes, we performed differential expression analysis between α*-MHC-MerCreMer*^*+/−*^*TOP2B*^*LSL/−*^ cardiomyocytes and control α*-MHC-MerCreMer*^*+/−*^ cardiomyocytes. We identified robust transcriptional reprogramming characterized by marked upregulation of the extracellular matrix (ECM) and structural remodeling genes. Among the most significantly upregulated genes were Myh8, Myh7, Col9a2, etc., many of which are associated with fibrosis, matrix deposition, and pathologic remodeling. Several fetal or stress-associated cardiac genes, including Nppa and Myh7, were strongly induced, consistent with activation of a maladaptive remodeling program (Fig. 4E and F; Supplementary Table S3). In contrast, a broad set of genes involved in cardiomyocyte contractile function and calcium handling and mitochondrial energy metabolism were significantly downregulated. This included canonical adult cardiomyocyte markers and excitation–contraction coupling genes such as Myh6, Tnni3, and Myl2. In parallel, multiple genes required for mitochondrial respiration and ATP production were suppressed, including UCP3 and Slc25a11, consistent with impaired oxidative phosphorylation and reduced bioenergetic capacity (Fig. 4E and F; Supplementary Table S3). GO enrichment analysis corroborated these patterns. Upregulated genes were enriched for biological processes related to collagen fibril organization, external encapsulating structure organization, and other remodeling-associated programs (Supplementary Fig. S3A). Conversely, downregulated genes were strongly enriched for mitochondrial pathways, including electron transport chain, NADH dehydrogenase complex assembly, oxidative phosphorylation, aerobic/cellular respiration, and ATP biosynthetic process, supporting a coordinated suppression of mitochondrial respiration in TOP2B-activated cardiomyocytes (Supplementary Fig. S3B). MYH6 (fast-twitch, high ATPase activity) to MYH7 (slow-twitch, low ATPase activity) myosin protein shift (15) and NPPA upregulation (16) are well-documented molecular signature in heart failure. UCP3 is a mitochondrial protein that plays a significant role in regulating energy metabolism and mitigating oxidative stress within cardiac cells (17, 18) and was found dramatically downregulated in the AIC rat model (19). Considering multiple KO and transgenic models, including cardiomyocyte-specific P53 KO (20) and C-MYC transgenic mice (21, 22), none exhibited acute heart failure. This strongly suggests that not all key gene overexpression or dysregulation inherently induces cardiac dysfunction. Therefore, the cardiotoxicity observed in our model is not a general consequence of gene dysregulation but rather a specific effect of TOP2B overexpression. The most attractive feature of our TOP2B transgenic model is that it mechanistically recapitulates AIC appearances seen in patients with AIC in the clinic. Together, these data show that TOP2B activation drives cardiomyocytes toward a remodeling/stress state characterized by ECM/fibrotic gene induction and concomitant loss of adult contractile and mitochondrial oxidative metabolism programs.

### Development of hTOP2B overexpression mouse models for chronic heart failure

**Figure 5.**
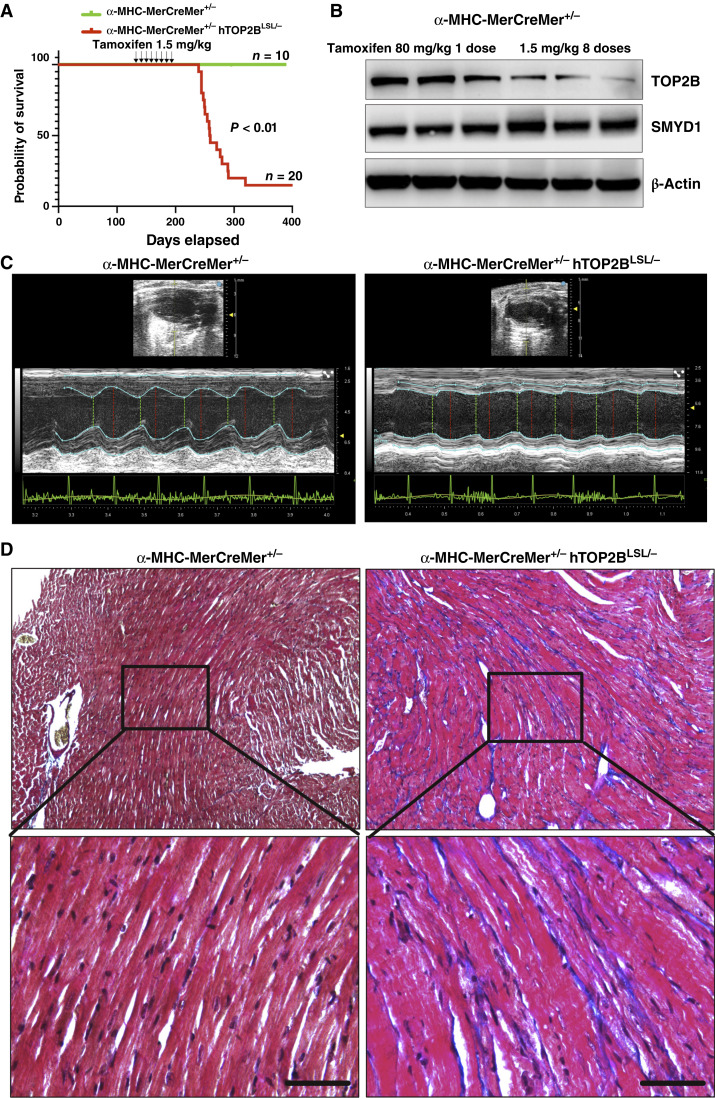
Chronic hTOP2B overexpression in cardiomyocytes induces chronic heart disease. **A,** Kaplan–Meier survival analysis of α-MHC-MerCreMer^+/−^ hTOP2B^LSL/−^ (red line) and control α-MHC-MerCreMer^+/−^ (green line) mice following tamoxifen (1.5 mg/kg) administration once per week for 8 weeks in 100-day-old mice. 17 transgenic mice (*n* = 20) exhibited significant mortality within 200 days, whereas control mice (*n* = 10) survived throughout the study (*P* < 0.01). Arrows indicate tamoxifen administration time points. **B** and **C,** Representative echocardiography images of control (α-MHC-MerCreMer^+/−^, left) and transgenic (α-MHC-MerCreMer^+/−^ hTOP2B^LSL/−^, right) mice. M-mode echocardiograms show LV chamber dilation and wall thinning in transgenic mice, consistent with heart failure. **D,** Masson’s Trichrome staining of heart tissue sections from α-MHC-MerCreMer^+/−^, TOP2B^LSL/−^ transgenic mice (*n* = 3) and α-MHC-MerCreMer^+/−^ control mice after last dose of tamoxifen treatment for 120 days.

Most doxorubicin regimens involve multiple doses, as early clinical experience in the 1970s demonstrated that single high-dose administration was associated with severe toxicity and a high risk of acute heart failure (23). To establish a mechanistically relevant mouse model for AIC and facilitate future therapeutic interventions, we generated α*-MHC-MerCreMer*^*+/−*^*hTOP2B*^*LSL/−*^ transgenic mice with tamoxifen-inducible hTOP2B overexpression in cardiomyocytes. To establish a chronic heart failure model, we administered 1.5 mg/kg/week tamoxifen for eight times (Fig. 5A). This regimen resulted in progressive cardiac deterioration, with 80% mortality by day 300, whereas all control mice (*n* = 10) survived (*P* < 0.01). The delayed yet inevitable disease progression closely mirrors chronic heart failure in patients undergoing prolonged anthracycline therapy with relatively chronic TOP2b overexpression compared with the 80 mg/kg single-dose regimen (Fig. 5B). To assess the functional consequences of hTOP2B overexpression on cardiac structure and function, transthoracic echocardiography was performed. Representative M-mode echocardiograms revealed severe LV dysfunction in α*-MHC-MerCreMer*^*+/−*^*hTOP2B*^*LSL/−*^ mice compared with controls (Fig. 5C; Supplementary Table S4). The transgenic mice exhibited LV wall thinning, chamber dilation, and heart fibrosis (Fig. 5D), indicative of progressive heart failure, whereas control mice maintained normal cardiac morphology and function. These data collectively suggest that hTOP2B overexpression in cardiomyocytes drives cardiac remodeling and progression to heart failure. These findings demonstrate that hTOP2B overexpression in cardiomyocytes is sufficient to drive anthracycline-like cardiotoxicity, reinforcing its critical role in AIC pathogenesis. The α*-MHC-MerCreMer*^*+/−*^*hTOP2B*^*LSL/−*^ mouse model provides a mechanism-based platform for evaluating novel cardioprotective strategies, including TOP2B-targeted therapies, epigenetic modulators, and cardioprotective agents aimed at mitigating anthracycline-induced heart failure.

To determine whether doxorubicin further exacerbates cardiac dysfunction in the setting of cardiomyocyte-specific TOP2B overexpression, we performed echocardiographic analysis in 1.5 mg/kg one dose/week tamoxifen-induced α*-MHC-MerCreMer*^*+/−*^*hTOP2B*^*LSL/−*^ mice with or without doxorubicin treatment (*n* = 7 per group). Compared with α*-MHC-MerCreMer*^*+/−*^*hTOP2B*^*LSL/−*^ mice without doxorubicin, single low-dose (5 mg/kg) doxorubicin-treated α*-MHC-MerCreMer*^*+/−*^*hTOP2B*^*LSL/−*^ mice exhibited a significant reduction in cardiac output (15.41 ± 0.86 vs. 18.92 ± 0.96 mL/minute, *P* = 0.0425) in echocardiography at 72 hours and a marked decrease in ejection fraction (38.89% ± 1.64% vs. 54.27% ± 1.55%, *P* < 0.0001; Supplementary Table S5). Stroke volume and heart rate showed a decreasing trend but did not reach statistical significance. LV mass and corrected LV mass were not significantly altered between groups. These findings demonstrate that doxorubicin markedly aggravates systolic dysfunction in TOP2B-overexpressing hearts, supporting a central role of TOP2B in mediating AIC.

### TOP2B binds to SMYD1 in cardiomyocytes

**Figure 6.**
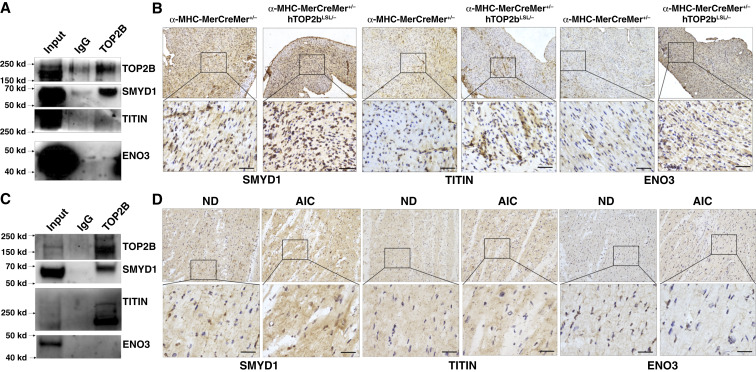
TOP2B binds SMYD1. **A,** Western blot analysis after immunoprecipitation (IP) of TOP2B in transgenic mice, probed for SMYD1, TITIN, and ENO3. **B,** IHC analysis showing the localization and expression levels of SMYD1, TITIN, and ENO3 in tissue sections from TOP2B transgenic mice. **C,** Western blot analysis after IP of TOP2B in patient samples with AIC, probed for SMYD1, TITIN, and ENO3. **D,** IHC analysis showing the localization and expression levels of SMYD1, TITIN, and ENO3 in tissue sections from AIC patient samples.

To explore the mechanism underlying the induction of AIC disease by TOP2B, we performed an TOP2B pulldown followed by LC-MS proteomic analysis using TOP2B antibody in the heart tissue from TOP2B transgenic (α*-MHC-MerCreMer*^*+/−*^, *TOP2B*^*LSL/−*^) mice. The most abundant cardiomyocytes or muscle-specific expression proteins were from TOP2B pulldown proteins, including SMYD1, TTN (Titin), and ENO3 (Supplementary Table S6). We focus on the SMYD1 because of its verified interaction with TOP2B by intraperitoneal Western blot and IHC using the TOP2B transgenic mouse model (Fig. 6A) and AIC patients’ sample (Fig. 6B). The Su(Var)3–9, enhancer-of-zeste and trithorax (SET) and myeloid, nervy, and DEAF-1 (MYND) domain-containing (SMYD) proteins, named SMYD1, SMYD2, SMYD3, SMYD4, and SMYD5, are enzymes that catalyze methylation of histone and nonhistone substrates, thereby playing a key role in gene expression regulation in many biological contexts, such as muscle development and physiology, hematopoiesis, and many types of cancer (24). SMYD1 is specifically expressed in cardiomyocytes and regulates histone-lysine N-methyltransferase activity (25). It is involved in the positive regulation of myoblast differentiation and myotube differentiation. Although SMYD1 is usually located in the cytoplasm, it is active in the nucleus (25). Furthermore, mutations in SMYD1 are associated with congenital myopathies and dilated cardiomyopathy (26, 27). These mutations disrupt the normal function of the protein, leading to muscle and cardiac abnormalities which mimics the AIC phenotype, raising the potential existence of shared pathophysiologic mechanisms between these two conditions. This finding underscores the complexity of both SMYD1-related diseases and AIC while hinting at potential commonalities in their underlying mechanisms. Mutations or dysregulation of SMYD1 can lead to various cardiac disorders (28), and their clinical presentation may overlap with AIC, suggesting that these diseases share pathophysiologic mechanisms implies that there may be common molecular pathways or cellular processes involved in both SMYD1-related diseases and AIC. This insight opens exciting avenues for research, as identifying these shared mechanisms could provide valuable insights into the underlying causes and potential therapeutic targets.

### TOP2B binds the S-domain of SMYD1

**Figure 7.**
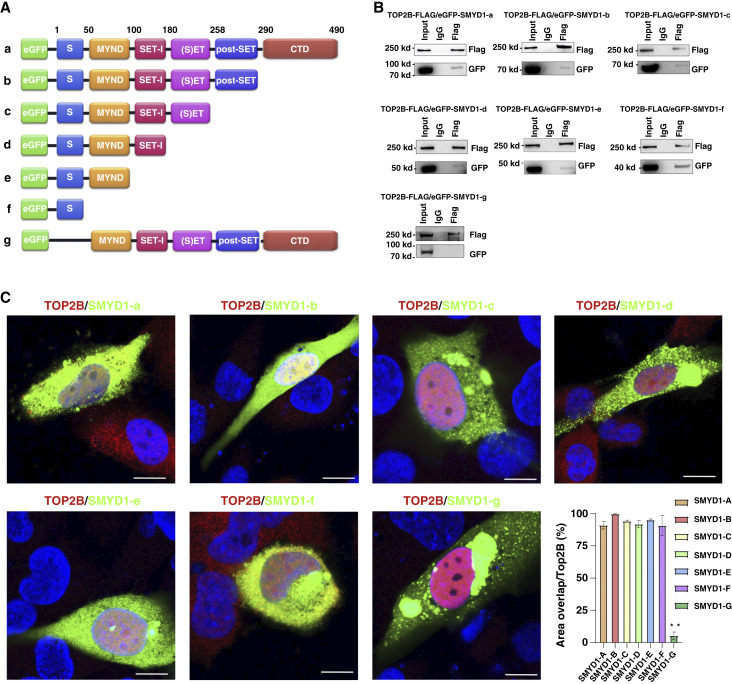
TOP2B binds to the S domain of SMYD1. **A,** Schematic diagram of SMYD1 truncation mutants fused to an N-terminal eGFP tag. Seven constructs were generated: a, full-length SMYD1 (residues 1–490); b, residues 1–290 lacking the C-terminal domain (CTD); c, residues 1–258 lacking post-SET and CTD; d, residues 1–180 lacking SET, post-SET, and CTD; e, residues 1–100 containing only the MYND domain; f, residues 1–50 containing only the S domain; and g, residues 100–490 lacking the S domain. **B,** Coimmunoprecipitation (co-IP) analysis of interactions between FLAG-tagged TOP2B and each eGFP-tagged SMYD1 truncation mutant in HEK293 cells. Anti-FLAG antibody was used for IP, and bound proteins were detected by immunoblotting with anti-FLAG and anti-GFP antibodies. Strong interaction was observed for constructs containing the S domain (constructs a–f), whereas the mutant lacking the S domain (g) showed loss of interaction. **C,** Representative confocal images of AC16 cells coexpressing FLAG-TOP2B and each eGFP-SMYD1 truncation mutant. Immunofluorescence staining of TOP2B (red), eGFP-SMYD1 (green), and nuclei (blue, DAPI). Colocalization of TOP2B and SMYD1 was evident for constructs containing the S domain (a–f), whereas the mutant lacking the S domain (g) exhibited no colocalization with TOP2B. **, *P* < 0.01; Scale bar, 10 μm.

To identify the domain of SMYD1 responsible for interacting with TOP2B, plasmids expressing different fragments of SMYD1 fused to an N-terminal eGFP tag were generated (Fig. 7A). These fragments included the S-domain, SET domain, MYND domain, and other relevant regions. The constructs were designed to facilitate targeted investigation of domain-specific interactions. FLAG-tagged TOP2B and eGFP-tagged SMYD1 fragments were coexpressed in AC16 human cardiomyocyte cells. Immunoprecipitation was performed using an anti-FLAG antibody to pull down FLAG-TOP2B and its associated binding partners. The resulting precipitates were analyzed via Western blot to detect interactions between TOP2B and the individual SMYD1 fragments. The results demonstrated that TOP2B specifically binds to the S-domain of SMYD1, as indicated by a strong signal in the immunoprecipitation assay (Fig. 7B). Other domains of SMYD1, including the SET and MYND domains, showed no binding to TOP2B. To further validate the interaction between TOP2B and SMYD1 and determine whether this interaction occurs within cells, we examined the subcellular localization of TOP2B and different SMYD1 fragments by immunofluorescence microscopy. AC16 human cardiomyocytes were cotransfected with constructs expressing TOP2B together with eGFP-tagged SMYD1 fragments. TOP2B was detected using anti-TOP2B FLAG antibody (red), whereas SMYD1 fragments were visualized through the eGFP signal (green), and nuclei were counterstained with DAPI (blue). Confocal imaging revealed clear colocalization of TOP2B with the SMYD1 fragment containing the S-domain, predominantly within the nucleus of cardiomyocytes. In contrast, fragments lacking the S-domain showed minimal or no detectable co-localization with TOP2B (Fig. 7C). Quantitative analysis of fluorescence overlap confirmed that the S-domain–containing constructs SMYD1 a-f exhibited significantly higher colocalization with TOP2B compared with SMYD1 S-domain deletion fragment-SMYD1-g. These results further support the biochemical findings from the coimmunoprecipitation assays and demonstrate that the S-domain mediates the intracellular interaction between TOP2B and SMYD1 in cardiomyocytes.

### TOP2B ASO prolongs survival in both TOP2B transgenic AIC mice and AIC mice

**Figure 8.**
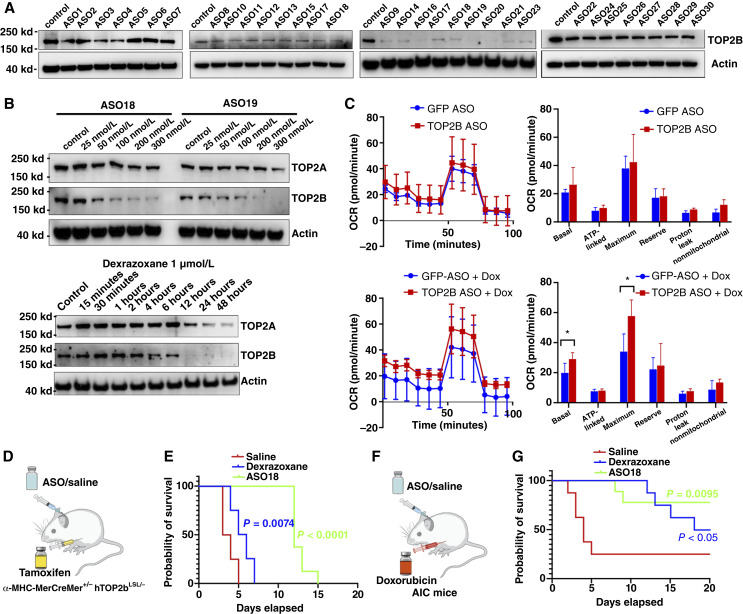
TOP2B ASO prevents cardiotoxicity in TOP2B transgenic mice and in an AIC mouse model. **A,** Western blot analysis showing suppression of TOP2B expression following transfection with TOP2B ASO in a cardiomyocyte cell line. **B,** Dose-dependent Western blot analysis of TOP2B ASO-18 and ASO-19 verifying their effects on TOP2B and TOP2A expression. **C,** Seahorse XF analysis of mitochondrial OCR in cells transfected with control GFP ASO or TOP2B ASO, with or without doxorubicin treatment. Key mitochondrial parameters measured include basal respiration, ATP-linked respiration, maximal respiration, reserve capacity, proton leak, and nonmitochondrial respiration. Data represent the mean ± SEM. Statistical significance was determined using an unpaired two-tailed Student *t* test; *, *P* < 0.05. **D,** Schematic of the TOP2B transgenic mouse heart failure model used for *in vivo* testing. **E,***In vivo* experiment showing that TOP2B ASO-18 treatment provides cardioprotective effects compared with dexrazoxane or saline in TOP2B transgenic mice (*n* = 8 for each group). **F,** Schematic of the AIC mouse model used for *in vivo* testing. **G,***In vivo* experiment showing that TOP2B ASO-18 treatment provides cardioprotective effects compared with dexrazoxane or saline in the AIC mouse model (*n* = 8 for each group).

TOP2B KO mice can tolerate lethal doses of doxorubicin (6), which supports the rationale for developing a TOP2B-specific ASO treatment for AIC. We screened 30 ASOs targeting human TOP2B mRNA in the human AC-16 cardiomyocyte cell line (Fig. 8A; Supplementary Table S7) and identified ASO-18 as the most effective candidate (Fig. 8B) for further proof-of-concept studies in both TOP2B transgenic AIC and AIC mouse models. To examine the role of TOP2B in regulating mitochondrial function in cardiomyocytes, we conducted Seahorse XF analysis in AC16 cells transfected with GFP ASO or TOP2B ASO. Under basal conditions, TOP2B ASO did not significantly change basal respiration, ATP-linked respiration, and maximal respiration compared with GFP ASO controls, reserve capacity, proton leak, and nonmitochondrial respiration (Fig. 8C). These results suggest that TOP2B depletion did not dysregulate the normal function of mitochondrial activity. Following doxorubicin treatment, notably, TOP2B ASO-transfected cells maintained significantly higher basal and maximal oxygen consumption respiration upon doxorubicin exposure compared with GFP ASO-transfected cells, indicating TOP2B depletion protected mitochondrial function under doxorubicin treatment. Together, these findings suggest that TOP2B knockdown preserved mitochondria function and confers protection against doxorubicin-induced mitochondrial injury (Fig. 8C).

In clinical settings, dexrazoxane is administered after doxorubicin to avoid compromising the efficacy of doxorubicin due to off-target effects on TOP2A. However, there is ongoing clinical trial leaded by Dr. Hui-Ming Chang in University of Arkansas for Medical Sciences to test the prevention efficiency by giving the dexrazoxane before giving the doxorubicin treatment. Because the half-life of dexrazoxane is 2 hours, 93.75% of dexrazoxane will be eliminated 8 hours (four half-lives) after administration. Indeed, dexrazoxane pretreatment 8 hours before doxorubicin provided complete protection against doxorubicin-induced cardiotoxicity in an animal model (29). Previous study had identified that ASO drug reaches peak levels in the heart within approximately 30 minutes (30). Considering that TOP2B upregulation starting as early as 15 minutes after treatment), we administered TOP2B ASO-18 30 minutes prior to tamoxifen or doxorubicin treatment. Our *in vivo* studies by delivery the ASO-18 to the mice demonstrate that ASO-18 significantly extends survival in TOP2B transgenic mice (Fig. 8D), increasing the mean survival time from 5 to 14 days (Fig. 8E); in comparison, dexrazoxane only showed slightly increasing of survival (*P* = 0.0074). In another AIC mouse model, this pretreatment approach (Fig. 8F) markedly improved survival in the AIC mouse model. Whereas approximately 75% of saline-treated mice succumbed to doxorubicin-induced heart failure by day 20, pretreatment with TOP2B ASO-18 reduced mortality to ∼25%, corresponding to ∼75% survival. In comparison, dexrazoxane provided only partial protection, improving survival to approximately 50% by day 20. Thus, TOP2B ASO-18 demonstrated superior cardioprotective efficacy compared with dexrazoxane in this model (Fig. 8G).

## Discussion

The mechanism underlying anthracycline-related cardiotoxicity has long been attributed to off-target effects on TOP2B in cardiomyocytes. On a molecular level, doxorubicin is thought to intercalate with DNA to prevent the final religation of DNA strands after TOP2B-mediated unwinding during replication and transcription, thus triggering widespread dsDNA-break DNA damage repair pathway, which can lead to both apoptosis and senescence. Depletion of TOP2B *in vitro* and *in vivo* are effective in mitigating doxorubicin cardiotoxicity in preclinical settings, and the efficacy of dexrazoxane in the clinical setting, though initially attributed to iron chelation, is now thought to also be an result of reduced TOP2B levels within tissues (6, 31, 32). The pathophysiologic features of AIC include myocyte hypertrophy, accumulation of lipofuscin and other markers of senescence, and tissue remodeling with increased fibrosis (33–35). The preservation of heart function observed in TOP2B KO mice provides a strong rationale for developing further TOP2B-targeted preventive therapies for patients with AIC (6). Patients with AIC also show a significant increase of β1-adrenergic receptors at the plasma membrane (36) which provides a platform for TOP2B-targeting drug delivery to cardiomyocytes. However, it remains to be demonstrated whether the TOP2B mechanism is mediated through genotoxic TOP2B–DNA complexes or an intrinsic function of the TOP2B protein, which we find is upregulated upon doxorubicin exposure. Accumulation of TOP2B after doxorubicin may indicate stalled TOP2B–DNA complexes, and it has been hypothesized that degradation of these complexes by the proteasome then leads to the DNA damage response. However, using a powerful transgenic mouse approach, we demonstrate for the first time that TOP2B upregulation, independently of doxorubicin exposure, is sufficient to induce a cardiotoxicity phenotype that mimics AIC, casting doubt on the veracity of the DNA-dependent hypothesis. For the first time, we investigate AIC at the spatial transcriptome level to match morphologic findings and RNA expression, along with novel MRI technology to bridge imaging findings with molecular mechanisms. Our data add to recent studies that elucidate the dual functions of certain DNA repair proteins which, when upregulated in response to catastrophic DNA damage or cellular stress, in fact trigger novel mechanisms of cell death (Supplementary Fig. S4).

We identify a new molecular role for TOP2B, specifically its binding to SMYD1 in postmitotic cardiomyocytes. This discovery contrasts with its well-known functions in the DNA repair pathway and sheds light on an epigenetic regulatory pathway that may be central to its cardiotoxic effects. SMYD1 is a histone methyltransferase that has been characterized in a variety of contexts and is widely reported to be protective against cardiac hypertrophy and disease. A number of cases of severe congenital cardiomyopathy or unexplained hypertrophic and noncompaction cardiomyopathy in adults have been attributed to SMYD1 mutations (26, 27, 37). Global Smyd-1 KO in mice results in embryonic lethality due to disrupted maturation of RV myocytes (38). Smyd-1 cardiomyocyte specific KO adult mice develop hypertrophic cardiomyopathy and heart failure, a phenotype attributed to histone demethylation and downregulation of PGC-1a, a master regulator of cardiac energetics (39, 40). At the current time, our findings are consistent with a model in which TOP2B upregulation compromises SMYD1 functions, resulting in cardiomyocyte hypertrophy, cardiomyopathy, and congestive heart failure. Functional characterization of TOP2B in this context will serve as a prototype, laying the foundation for future studies that seek to unveil unknown functions of known genes, particularly in the context of doxorubicin therapy.

A hallmark of pathologic cardiac remodeling in cardiomyopathy is the reactivation of a fetal gene program characterized by a switch in myosin heavy chain isoform expression from MYH6 (α-MHC) to MYH7 (β-MHC). Whereas MYH6 predominates in the adult myocardium and supports faster contractile kinetics, MYH7, a slower and more energy-efficient isoform, becomes upregulated during chronic stress, contributing to impaired systolic performance and disease progression. This isoform shift is tightly regulated by cardiac transcriptional and epigenetic mechanisms, among which SMYD1, a cardiac-specific histone methyltransferase, plays a critical role. SMYD1 directly promotes MYH6 transcription by maintaining an active chromatin state at its promoter and supporting sarcomeric gene expression. In our study, we identified TOP2B as a novel upstream disruptor of this regulatory axis. Specifically, we found that pathological overexpression of TOP2B—either induced by anthracycline exposure or modeled via transgenic expression—physically interacts with and functionally impairs SMYD1 in cardiomyocytes. This disruption leads to suppressed MYH6 expression and enhanced MYH7 upregulation, exacerbating the maladaptive transcriptional reprogramming typical of failing hearts. These findings reveal a previously unrecognized TOP2B–SMYD1–MYH6/MYH7 axis that may contribute to the molecular pathogenesis of anthracycline-induced and stress-related cardiomyopathy. Therapeutically targeting this pathway may offer new opportunities to preserve contractile gene expression and cardiac function in high-risk patients.

Importantly, we also demonstrate that targeted inhibition of TOP2B using ASO therapy markedly protects against doxorubicin-induced cardiotoxicity and significantly improves survival in preclinical models. Compared with the current clinically approved cardioprotective agent dexrazoxane, TOP2B ASO therapy provides more effective protection in our experimental models, suggesting that direct suppression of TOP2B represents a more precise and potentially superior preventive strategy. Collectively, these findings establish TOP2B as a critical driver of AIC and highlight TOP2B-targeted ASO therapy as a promising prophylactic approach for patients undergoing anthracycline chemotherapy.

## Supplementary Material

Supplementary Figure S1 Figure S1. Doxyrubicin modestly increases TOP2B mRNA expression in mouse heart tissue.

Supplementary Figure S2 Figure S2. Doxorubicin significantly increases TOP2B protein expression in mouse heart tissue.

Supplementary Figure S3 Figure S3. Gene Ontology(GO) Biological Process enrichment analysis of differentially expressed genes.

Supplementary Figure S4 Figure S4. Proposed mechanism of anthracycline-induced cardiotoxicity and prevention by TOP2B ASO therapy.

Supplementary Table S1 Table S1. Baseline characteristics for study population.

Supplementary Table S2 Table S2. Echocardiograph data analysis in acute heart failure mouse model after tamoxifen was given 3 days.

Supplementary Table S3 Table S3. Differentially expressed genes identified by RNA-seq analysis in cardiomyocytes from tamoxifen-induced _-MHC-MerCreMer+/_hTOP2B LSL/_ mice mice compared with _-MHC-MerCreMer+/_ control mice.

Supplementary Table S4 Table S4. Echocardiograph data analysis in chronic heart failure mouse model after tamoxifen was given 150 days.

Supplementary Table S5 Table S5. Echocardiograph data analysis in chronic heart failure mouse model after tamoxifen with or without doxorubicin treatment.

Supplementary Table S6 Table S6. Analyses of TOP2B pull down proteins by LS/MS. (LTS= low tissue specificity)

Supplementary Table S7 Table S7. TOP2B ASO sequences.

Supplementary Video 1 Supplementary Video 1

Supplementary Video 2 Supplementary Video 2

Supplementary Video 3 Supplementary Video 3

## Data Availability

The RNA sequencing data generated in this study have been deposited in the Gene Expression Omnibus under accession number GSE319842. The mass spectrometry proteomics data have been deposited to the ProteomeXchange Consortium via the PRIDE partner repository with the dataset identifier PXD077277 and 10.6019/PXD077277. All other data generated or analyzed during this study are available from the corresponding author upon reasonable request.
