# Supplementary figures and images for: Novel Mechanism of and Therapeutic Approach for Anthracycline-Induced Cardiotoxicity
Source: Cancer Res Commun. 2026 Jun 1;6(6):1261–77. doi: 10.1158/2767-9764.CRC-25-0511 (PMC13223395; doi:10.1158/2767-9764.CRC-25-0511)

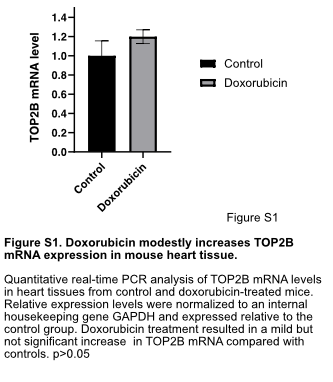

Supplement: Supplementary Figure S1 — Figure S1. Doxyrubicin modestly increases TOP2B mRNA expression in mouse heart tissue. [file crc-25-0511_supplementary_figure_s1_suppsf1.png]

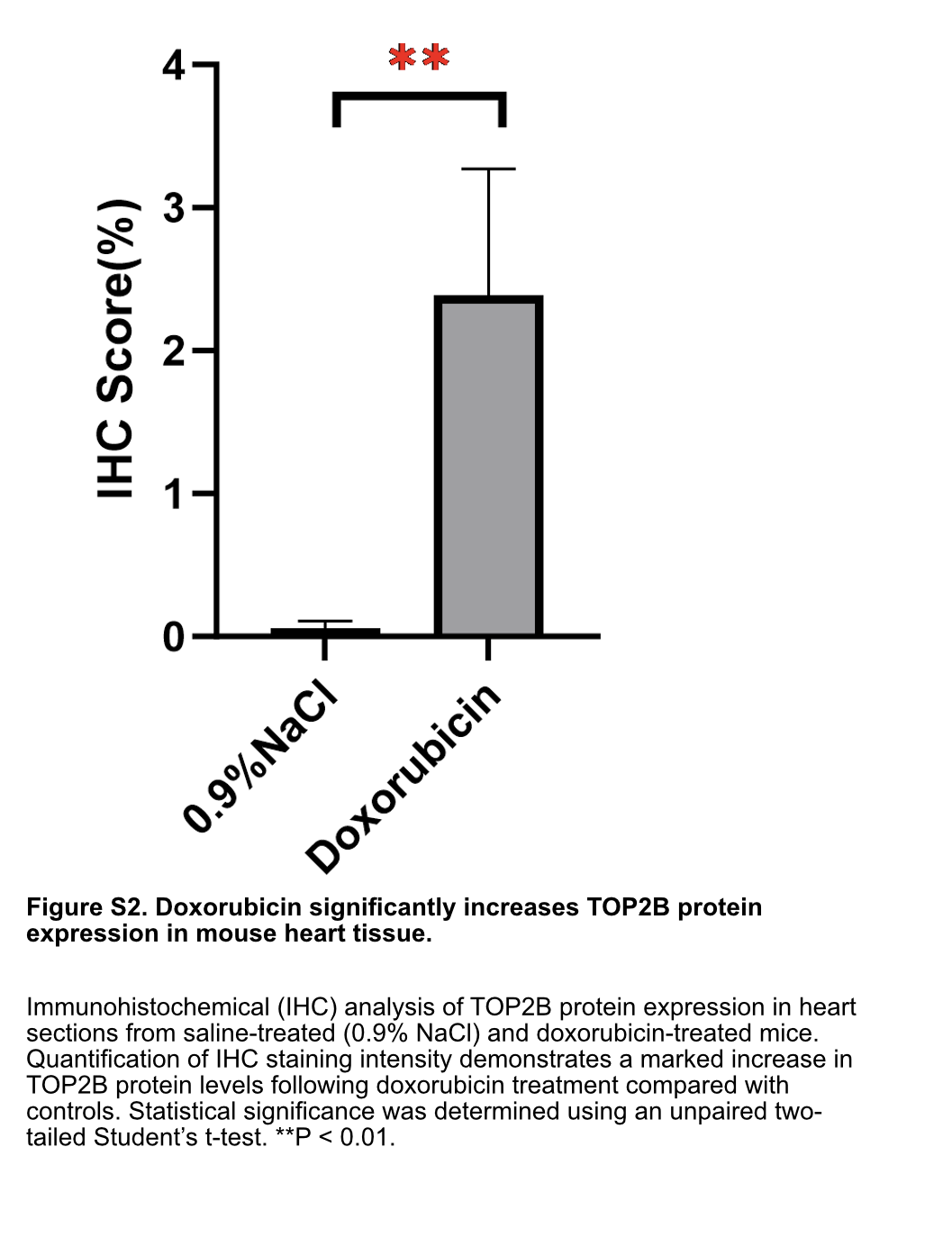

Supplement: Supplementary Figure S2 — Figure S2. Doxorubicin significantly increases TOP2B protein expression in mouse heart tissue. [file crc-25-0511_supplementary_figure_s2_suppsf2.png]

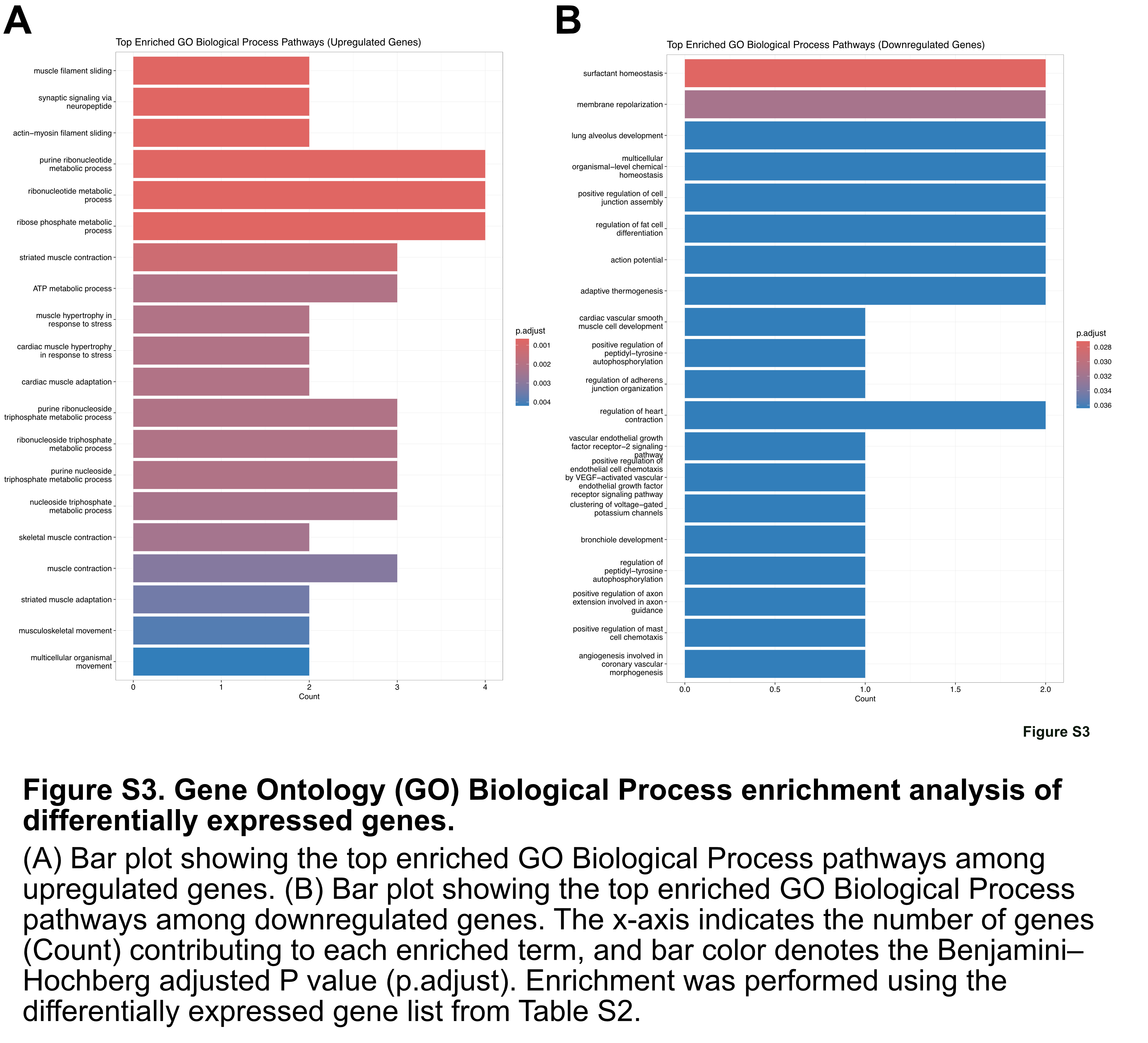

Supplement: Supplementary Figure S3 — Figure S3. Gene Ontology(GO) Biological Process enrichment analysis of differentially expressed genes. [file crc-25-0511_supplementary_figure_s3_suppsf3.png]

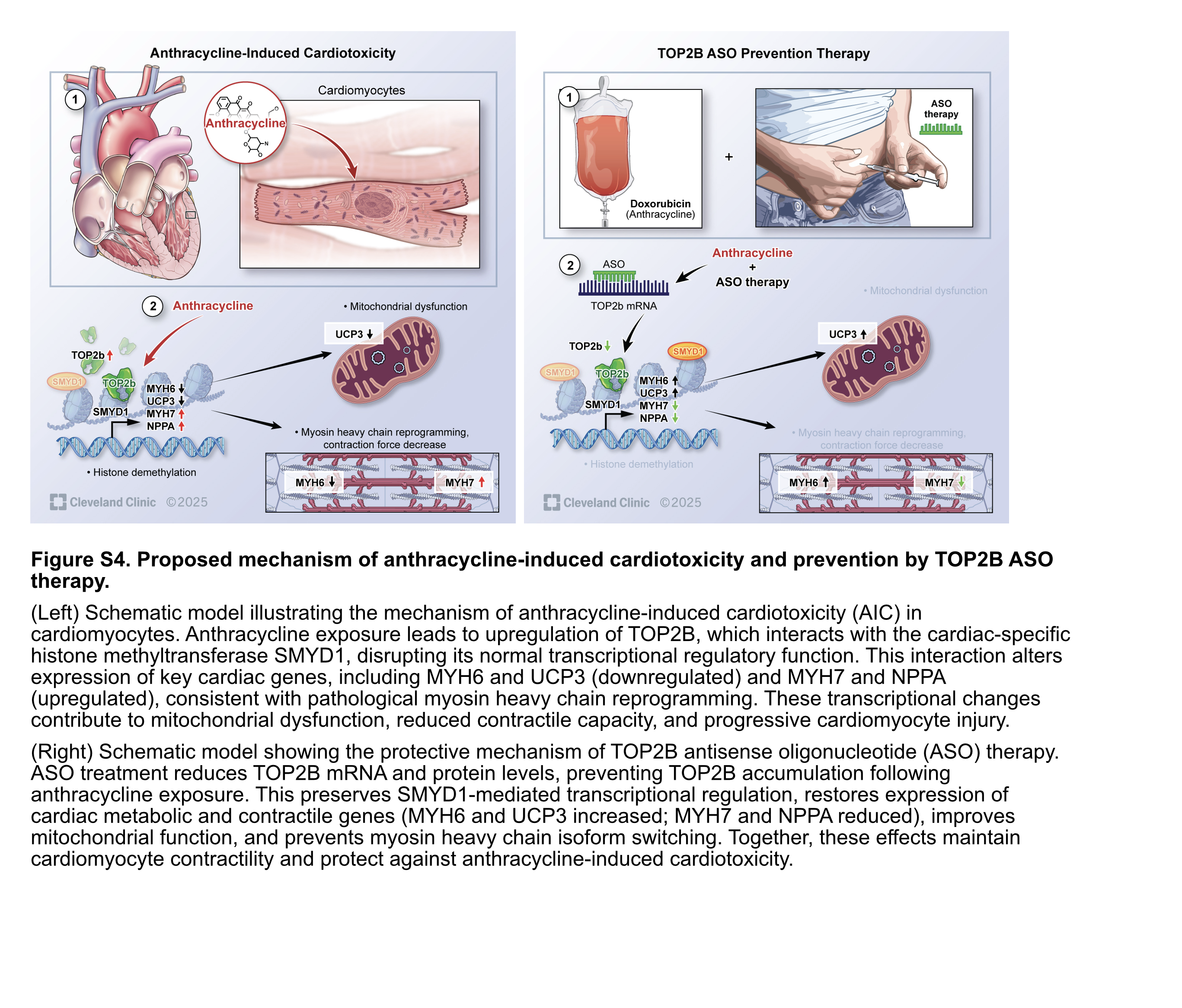

Supplement: Supplementary Figure S4 — Figure S4. Proposed mechanism of anthracycline-induced cardiotoxicity and prevention by TOP2B ASO therapy. [file crc-25-0511_supplementary_figure_s4_suppsf4.png]
