# Supplementary material for: Novel Mechanism of and Therapeutic Approach for Anthracycline-Induced Cardiotoxicity
Source: Cancer Res Commun. 2026 Jun 1;6(6):1261–77. doi: 10.1158/2767-9764.CRC-25-0511 (PMC13223395; doi:10.1158/2767-9764.CRC-25-0511)
Supplement: Supplementary Table S1 — Table S1. Baseline characteristics for study population. [file crc-25-0511_supplementary_table_s1_suppst1.docx]

# Table S1. Baseline characteristics for study population.

| ID | Age | Sex | Race | Cancer Type | Timing from last dose to HF diagnosis | Timing from last dose to transplant | Concomitant Radiation | Hypertension | Diabetes Mellitus | Chronic Kidney Disease |
| --- | --- | --- | --- | --- | --- | --- | --- | --- | --- | --- |
| TM1 | 34 | Male | White | Rhabdomyosarcoma | 28.5 years | 31.5 years | Yes | No | No | Yes |
| TM2 | 27 | Male | White | Rhabdomyosarcoma | 6.5 years | 19.5 years | Yes | Yes | No | Yes |
| TM3 | 49 | Male | White | Hodgkin's Lymphoma | 13.5 years | 16.5 years | No | No | Yes | Yes |
| TF1 | 48 | Female | Black | Breast cancer | 1 year | 2 years | Yes | No | No | No |
| TF2 | 57 | Female | White | Breast cancer | 2.5 years | 2.5 years | Yes | No | No | No |
| TF3 | 68 | Female | White | Breast cancer | 14 years | 19 years | Yes | Yes | No | Yes |
| TF4 | 61 | Female | White | Breast cancer | < 1 year | 15 years | Yes | Yes | No | Yes |
| TF5 | 62 | Female | White | Breast cancer | 3 years | 21 years | No | Yes | No | Yes |
| TF6 | 64 | Female | White | Breast cancer | 2 years | 10 years | Yes | No | Yes | No |
| TF7 | 65 | Female | White | Breast cancer | 1 year | 16 years | Yes | No | Yes | Yes |
| TF8 | 38 | Female | White | Breast cancer | 2.5 years | 32.5 years | No | No | Yes | Yes |
| TF9 | 63 | Female | Black | Breast cancer | < 1 year | 7 years | Yes | No | Yes | Yes |
| TF10 | 65 | Female | Black | Breast cancer | 4.5 years | 12.5 years | Yes | Yes | No | Yes |
| TF11 | 63 | Female | White | Leiomyosarcoma | 17 years | 21 years | Yes | No | No | Yes |
| TF12 | 63 | Female | White | Breast cancer | 24 years | 24 years | Yes | No | Yes | No |
| TF13 | 58 | Female | White | Non-Hodgkin's lymphoma | 16.5 years | 16.5 years | No | No | No | No |
| TF14 | 56 | Female | Multi-racial | Breast cancer | 4 years | 16 years | Yes | Yes | Yes | Yes |
