# Supplementary material for: Novel Mechanism of and Therapeutic Approach for Anthracycline-Induced Cardiotoxicity
Source: Cancer Res Commun. 2026 Jun 1;6(6):1261–77. doi: 10.1158/2767-9764.CRC-25-0511 (PMC13223395; doi:10.1158/2767-9764.CRC-25-0511)
Supplement: Supplementary Table S2 — Table S2. Echocardiograph data analysis in acute heart failure mouse model after tamoxifen was given 3 days. [file crc-25-0511_supplementary_table_s2_suppst2.docx]

**Table S2. Echocardiograph data analysis in acute heart failure mouse model after tamoxifen was given 3 days.**

| **Pressure-volume**  **Loop parameters** | **α-MHC-MerCreMer^+/−^ mice**  **(Mean±SEM, n=10)** | **α-MHC-MerCreMer^+/−^hTOP2B ^LSL/−^** **mice**  **(Mean±SEM, n=10)** | **p-Value** |
| --- | --- | --- | --- |
| Cardiac output(mL/min) | 20.39±1.75 | 4.99±0.75 | <0.0001 |
| Stroke volume (μL) | 43.47±3.67 | 12.02±1.70 | <0.0001 |
| Heart rate (bpm) | 468.73±7.69 | 414.79±16.99 | 0.0097 |
| Ejection fraction (%) | 57.30±3.25 | 11.48±1.79 | <0.0001 |
| LV Mass | 153.18±10.56 | 135.57±11.78 | 0.2805 |
| LV Mass Cor | 122.54±8.45 | 108.46±9.42 | 0.2805 |
