# Supplementary material for: Novel Mechanism of and Therapeutic Approach for Anthracycline-Induced Cardiotoxicity
Source: Cancer Res Commun. 2026 Jun 1;6(6):1261–77. doi: 10.1158/2767-9764.CRC-25-0511 (PMC13223395; doi:10.1158/2767-9764.CRC-25-0511)
Supplement: Supplementary Table S4 — Table S4. Echocardiograph data analysis in chronic heart failure mouse model after tamoxifen was given 150 days. [file crc-25-0511_supplementary_table_s4_suppst4.docx]

**Table S4. Echocardiograph data analysis in chronic heart failure mouse model after tamoxifen was given 150 days.**

| **Pressure-volume** | **α-MHC-MerCreMer^+/−^ mice** | **α-MHC-MerCreMer^+/−^hTOP2B ^LSL/−^** **mice** | **p-Value** |
| --- | --- | --- | --- |
| **Loop parameters** | **(Mean±SEM,n=8)** | **(Mean±SEM,n=20)** |  |
| **Cardiac output(mL/min)** | 19.16±1.02 | 13.31±0.74 | 0.0001 |
| **Stroke volume (μL)** | 38.33±1.88 | 27.03±1.46 | 0.0002 |
| **Heart rate (bpm)** | 499.70±9.80 | 492.60±8.86 | 0.6150 |
| **Ejection fraction (%)** | 63.22±4.14 | 35.74±3.08 | <0.0001 |
| **LV Mass** | 125.83±8.28 | 105.45±6.15 | 0.0612 |
| **LV Mass Corrected** | 100.66±6.63 | 84.36±4.92 | 0.0612 |
