# Supplementary material for: Novel Mechanism of and Therapeutic Approach for Anthracycline-Induced Cardiotoxicity
Source: Cancer Res Commun. 2026 Jun 1;6(6):1261–77. doi: 10.1158/2767-9764.CRC-25-0511 (PMC13223395; doi:10.1158/2767-9764.CRC-25-0511)
Supplement: Supplementary Table S5 — Table S5. Echocardiograph data analysis in chronic heart failure mouse model after tamoxifen with or without doxorubicin treatment. [file crc-25-0511_supplementary_table_s5_suppst5.docx]

**Table S5. Echocardiograph data analysis in chronic heart failure mouse model after tamoxifen with or without doxorubicin treatment.**

| Pressure-volume  Loop parameters | α-MHC-MerCreMer^+/-^ hTOP2B^LSL/-^mice wo doxorubicin (Mean±SEM, n=7) | | α-MHC-MerCreMer^+/-^ hTOP2B^LSL/-^mice with doxorubicin  (Mean±SEM, n=7) | p-Value |
| --- | --- | --- | --- | --- |
| Cardiac output(mL/min) | | 18.92±0.96 | 15.41±0.86 | 0.0425 |
| Stroke volume (μL) | | 36.04±1.53 | 31.01±1.52 | 0.0753 |
| Heart rate (bpm) | | 523.62±8.05 | 496.18±9.38 | 0.0879 |
| Ejection fraction (%) | | 54.27±1.55 | 38.89±1.64 | <0.0001 |
| LV Mass | | 127.54±4.12 | 117.03±5.48 | 0.2234 |
| LV Mass Cor | | 102.03±3.29 | 93.63±4.38 | 0.2234 |
