# Supplementary material for: Novel Mechanism of and Therapeutic Approach for Anthracycline-Induced Cardiotoxicity
Source: Cancer Res Commun. 2026 Jun 1;6(6):1261–77. doi: 10.1158/2767-9764.CRC-25-0511 (PMC13223395; doi:10.1158/2767-9764.CRC-25-0511)
Supplement: Supplementary Table S6 — Table S6. Analyses of TOP2B pull down proteins by LS/MS. (LTS= low tissue specificity) [file crc-25-0511_supplementary_table_s6_suppst6.docx]

**Table. S6. Analyses of TOP2B pull down proteins by LS/MS.** (LTS= low tissue specificity)

| **ID** | **kDa** | **IgG** | **IP** | **IP/IgG** | **Tissue specificity** | **Disease** |
| --- | --- | --- | --- | --- | --- | --- |
| Cct2 | 57.4 | 0 | 26 | IP only | LTS |  |
| Eno3 | 47 | 0 | 25 | IP only | muscle, cardio | Glycogen Storage Disease Xiii, rhabdomyolysis |
| Unc45b | 103.6 | 0 | 20 | IP only | muscle, cardio |  |
| Nono | 54.5 | 0 | 19 | IP only | LTS |  |
| Npm1 | 32.5 | 0 | 19 | IP only | LTS |  |
| Plec | 533.9 | 0 | 18 | IP only | LTS |  |
| Hp1bp3 | 60.8 | 0 | 17 | IP only | LTS |  |
| Acad8 | 45 | 0 | 16 | IP only | LTS |  |
| Ilf2 | 43 | 0 | 15 | IP only | LTS |  |
| Plg | 90.7 | 0 | 15 | IP only | Live, kidney |  |
| Iqgap1 | 188.6 | 0 | 14 | IP only | LTS |  |
| Nedd4 | 102.6 | 0 | 14 | IP only | LTS |  |
| Cps1 | 164.5 | 0 | 14 | IP only | liver |  |
| TOP2B | 183.2 | 0 | 13 | IP only | LTS |  |
| Apob | 509.1 | 0 | 12 | IP only | liver, intestine |  |
| Tpp2 | 139.8 | 0 | 12 | IP only | LTS |  |
| Mfn1 | 83.7 | 0 | 12 | IP only | LTS |  |
| Cand1 | 136.2 | 0 | 12 | IP only | LTS |  |
| Flot2 | 47 | 0 | 12 | IP only | LTS |  |
| Hspa4 | 94.1 | 0 | 12 | IP only | LTS |  |
| Ppia | 18 | 0 | 12 | IP only | LTS |  |
| Smyd1 | 56.5 | 0 | 12 | IP only | Cardio, muscle | Distal Muscular Dystrophy and Epiphyseal Dysplasia |
| Slit3 | 167.6 | 0 | 11 | IP only | LTS |  |
| Speg | 354.1 | 0 | 11 | IP only | smooth muscle |  |
| Sqor | 50.3 | 0 | 11 | IP only | intestine |  |
| Calm2 | 16.8 | 0 | 11 | IP only | LTS |  |
| Arhgap1 | 50.4 | 0 | 11 | IP only | LTS |  |
| Aspn | 42.5 | 0 | 11 | IP only | muscle cell enriched | |
| Syncrip | 69.6 | 0 | 11 | IP only | LTS |  |
| Ttn | 3904.1 | 0 | 10 | IP only | Cardio, muscle | Congenital Myopathy With Cardiomyopathy |
| Nfs1 | 50.5 | 0 | 10 | IP only | LTS |  |
| Rock1 | 158.1 | 0 | 10 | IP only | LTS |  |
| Gfm1 | 83.5 | 0 | 10 | IP only | LTS |  |
| Uso1 | 106.9 | 0 | 10 | IP only | muscle cell enriched | |
| Tkt | 67.6 | 0 | 10 | IP only | bone marrow |  |
| Atad3 | 66.7 | 0 | 10 | IP only | LTS |  |
| Mrpl40 | 24.3 | 0 | 10 | IP only | LTS |  |
| Pacsin2 | 55.8 | 0 | 10 | IP only | LTS |  |
