# Supplementary material for: Novel Mechanism of and Therapeutic Approach for Anthracycline-Induced Cardiotoxicity
Source: Cancer Res Commun. 2026 Jun 1;6(6):1261–77. doi: 10.1158/2767-9764.CRC-25-0511 (PMC13223395; doi:10.1158/2767-9764.CRC-25-0511)
Supplement: Supplementary Table S7 — Table S7. TOP2B ASO sequences. [file crc-25-0511_supplementary_table_s7_suppst7.docx]

**Table S7. TOP2B ASO sequences.**

| TOP2B ASO No | Target sequence | Gapmer ASO Design |  |
| --- | --- | --- | --- |
| TOP2B-ASO-1 | TTTAACATTTTGTTCTTACA | mU*mG*mU*mA*mA*G*A*A*C*A*A*A*A*T*G*mU*mU*mA*mA*mA |  |
| TOP2B-ASO-2 | TTGTACATCTGGCTTATTTT | mA*mA*mA*mA*mU*A*A*G*C*C*A*G*A*T*G*mU*mA*mC*mA*mA |  |
| TOP2B-ASO-3 | GCAATGTTTAATTAAGTGCC | mG*mG*mC*mA*mC*T*T*A*A*T*T*A*A*A*C*mA*mU*mU*mG*mC |  |
| TOP2B-ASO-4 | ATGTGGGTGTATGATGAAGATG | MU*MC*MU*MU*MC*A*T*C*A*T*A*C*A*C*C*MC*MA*MC*MA*MT |  |
| TOP2B-ASO-5 | TTATTACGGTTTACGTAAGG | MC*MC*MU*MU*MA*C*G*T*A*A*A*C*C*G*T*MA*MA*MU*MA*MA |  |
| TOP2B-ASO-6 | TTGATACTGCAGCAGTAAAAG | MU*MU*MU*MU*MA*C*T*G*C*T*G*C*A*G*T*MA*MU*MC*MA*MA |  |
| TOP2B-ASO-7 | ACTGTCTGATTGGCTTGTAGA | MU*MC*MU*MA*MC*A*A*G*C*C*A*A*T*C*A*MG*MA*MC*MA*MG |  |
| TOP2B-ASO-8 | TCTTCGTCCTGATACATATAT | mA*mU*mA*mU*mA*mU*G*T*A*T*C*A*G*G*mA*mC*mG*mA*mA*mG*mA |  |
| TOP2B-ASO-9 | GTTAATGCTGCTGACAATAAA | mU*mU*mU*AmU*mU*mG*T*C*A*G*C*A*G*C*mA*mU*mU*mA*mA*mC |  |
| TOP2B-ASO-10 | GATCCTGAATCTAACATTATA | mU*mA*mU*mA*mA*mU*mG*T*T*A*G*A*T*T*C*mA*mG*mG*mA*mU*mC |  |
| TOP2B-ASO-11 | AGTTTATGTTCCTGCTTTAAT | mA*mU*mU*mA*mA*mA*mG*C*A*G*G*A*A*C*A*mU*mA*mA*mA*mC*mU |  |
| TOP2B-ASO-12 | AGCAGACATGGATGAATAATA | mU*mA*mU*mU*mA*mU*mU*mC*A*T*C*C*A*T*G*T*mC*mU*mG*mC*mU | |
| TOP2B-ASO-13 | CAACCAGATCTGTCCAAATTT | mA*mA*mA*mU*mU*mU*mG*G*A*C*A*G*A*T*C*mU*mG*mG*mU*mU*mG |  |
| TOP2B-ASO-14 | AGCAAATCAGCTTTGTAAATA | mU*AmU*mU*mU*mA*C*A*A*A*G*C*T*G*mA*mU*mU*mU*mG*mC*mU |  |
| TOP2B-ASO-15 | ATCCTGAACTGGGTGAAATTT | mA*mA*mA*mU*mU*mU*mC*A*C*C*C*A*G*T*T*mC*mA*mG*mG*mA*mU |  |
| TOP2B-ASO-16 | CTGGAGTGTACACTGATATTA | mC*mU*mG*mG*mA*GT*G*T*A*C*A*C*T*mG*mA*mU*mA*mU*mU*mA |  |
| TOP2B-ASO-17 | CTAAAGAAGCAAAGGAATATT | mA*mA*mU*mA*mU*mU*C*C*T*T*T*G*C*T*mU*mC*mU*mU*mU*mA*mG |  |
| TOP2B-ASO-18 | TCTCTTGTTGATGGCTTTAAA | mU*mU*mU*mA*mA*mA*G*C*C*A*T*C*A*A*mC*mA*mA*mG*mA*mG*mA |  |
| TOP2B-ASO-19 | TGCTGCAAGCCCTCGTTATAT | mA*mU*mA*mU*mA*mA*mC*G*A*G*G*G*C*T*T*mG*mC*mA*mG*mC*mA |  |
| TOP2B-ASO-20 | GTAGAGCCTGAGTGGTATATT | mA*mA*mU*mA*mU*AC*C*A*C*T*C*A*G*mG*mC*mU*mC*mU*mA*mC |  |
| TOP2B-ASO-21 | ATGCAGTCAGTGGTGAAATAT | mA*mU*mA*mU*mU*mU*C*A*C*C*A*C*T*G*mA*mC*mU*mG*mC*mA*mU |  |
| TOP2B-ASO-22 | ACAGAAACACAGTAGAAATTA | mU*mA*mA*mU*mU*mU*C*T*A*C*T*G*T*G*mU*mU*mU*mC*mU*mG*mU |  |
| TOP2B-ASO-23 | GAACTTGGACACAGGTATATA | mU*mA*mU*mA*mU*mA*C*C*T*G*T*G*T*C*mC*mA*mA*mG*mU*mU*mC |  |
| TOP2B-ASO-24 | ACTGACACAACTGTGAAATTT | mA*mA*mA*mU*mU*mU*C*A*C*A*G*T*T*G*mU*mG*mU*mC*mA*mG*mU |  |
| TOP2B-ASO-25 | ATGGGATGTCTGAAGAAATAT | mA*mU*mA*mU*mU*mU*mC*T*T*C*A*G*A*C*A*mU*mC*mC*mC*mA*mU |  |
| TOP2B-ASO-26 | ACCTTATGGCAGAAGAATAAT | mA*mU*mU*mA*mU*mU*mC*T*T*C*T*G*C*C*A*mU*mA*mA*mG*mG*mU |  |
| TOP2B-ASO-27 | GACTCCATCAGTTCCTATAAA | mU*mU*mU*mA*mU*mA*mG*G*A*A*C*T*G*A*T*mG*mG*mA*mG*mU*mC |  |
| TOP2B-ASO-28 | AGCAGCCGAAAGACCTAAATA | mU*mA*mU*mU*mU*mA*mG*G*T*C*T*T*T*C*G*mG*mC*mU*mG*mC*mU |  |
| TOP2B-ASO-29 | GATGATGATGATGACAATAAT | mA*mU*mU*mA*mU*mU*mG*T*C*A*T*C*A*T*C*mA*mU*mC*mA*mU*mC |  |
| TOP2B-ASO-30 | AGAAGATGATTCAGCTAAATT | mA*mA*mU*mU*mU*mA*mG*C*T*G*A*A*T*C*A*mU*mC*mU*mU*mC*mU |  |
